# Genomic Polymorphism Associated with the Emergence of Virulent Isolates of *Mycobacterium bovis* in the Nile Delta

**DOI:** 10.1038/s41598-019-48106-3

**Published:** 2019-08-12

**Authors:** Hazem F. M. Abdelaal, Daniel Spalink, Ali Amer, Howard Steinberg, Emad A. Hashish, Essam A. Nasr, Adel M. Talaat

**Affiliations:** 1 Department of Pathobiological Sciences, University of Wisconsin-Madison, Madison, WI USA; 2 Department of Ecosystem Science and Management, Texas A&M University, College Station, TX USA; 3 Animal Health Research Institute, Dokki, Giza Egypt; 4 Department of Clinical Pathology, Faculty of Veterinary Medicine, Zagazig University, Zagazig, Egypt; 5 Veterinary Serum and Vaccine Research Institute, Bacterial Diagnostics Research Department (Tuberculosis), Abbasia, Cairo Egypt

**Keywords:** Bacterial genetics, Pathogens, Infectious diseases

## Abstract

*Mycobacterium bovis* is responsible for bovine tuberculosis in both animals and humans. Despite being one of the most important global zoonotic disease, data related to the ecology and pathogenicity of bovine tuberculosis is scarce, especially in developing countries. In this report, we examined the dynamics of *M. bovis* transmission among dairy cattle in the Nile Delta of Egypt. Animals belonging to 27 herds from 7 governorates were tested by the Single Intradermal Comparative Skin Tuberculin (SICST), as a preliminary screen for the presence of bovine tuberculosis. Positive SICST reactors were identified in 3% of the animals spread among 40% of the examined herds. Post-mortem examination of slaughtered reactors confirmed the presence of both pulmonary and/or digestive forms of tuberculosis in > 50% of the examined animals. Targeted and whole-genome analysis of *M. bovis* isolates indicated the emergences of a predominant spoligotype (SB0268) between 2013–2015, suggesting a recent clonal spread of this isolate within the Nile Delta. Surprisingly, 2 isolates belonged to *M. bovis* BCG group, which are not allowed for animal vaccination in Egypt, while the rest of isolates belonged to the virulent *M. bovis* clonal complex European 2 present in Latin America and several European countries. Analysis of strain virulence in the murine model of tuberculosis indicated the emergence of a more virulent strain (MBE4) with a specific genotype. More analysis is needed to understand the molecular basis for successful spread of virulent isolates of bovine tuberculosis among animals and to establish genotype/phenotype association.

## Introduction

*Mycobacterium bovis* is the most common causative agent of bovine tuberculosis (bTB), an important infectious disease of cattle all over the world. The Office International des Epizooties (OIE) identified bTB as a list B transmissible disease of public health importance and of high impact on the international trade of animals and animal products^1^. Although national campaigns of “test and slaughter strategy” have reduced the incidence of the infection worldwide, bTB remains an important public health concern because of its zoonotic potential and re-emergence in animals and humans^2^. Bovine tuberculosis is a chronic, debilitating infection that infects a wide range of hosts including domesticated and wild animals^3^. Unlike *M. tuberculosis*, *M. bovis* has an unusually extensive host range including humans as recognized by the World Health Organization^4^ with a greater zoonotic potential in developing countries. *M. bovis* is also the progenitor of the Bacillus Calmette–Guérin (BCG), the only licensed tuberculosis vaccine and the gold standard for protection against childhood disseminated tuberculosis^5^. Despite its worldwide use for humans^5^, BCG is not approved for use in cattle vaccination in most of the world. In Egypt and other African countries, bTB represents a significant health and economic problem^6,7^ that requires further analysis on both genetic and genomic levels of *M. bovis*. For example, in Egypt the animal-level prevalence of bTB in cattle and buffaloes during the 1980s ranged between 6.9% and 26.2% then was reduced to 2.6% during the 1990s^6^. However, recent reports on the ecology, genotypes and virulence of *M. bovis* isolates circulating in Egypt are lacking and hence, the focus of this report.

*M. bovis* is a highly clonal pathogen^8^, where clonal complexes are defined based on chromosomal deletion, spoligotyping and MIRU-VNTR to define dynamics of disease transmission^9,10^. Recently, Whole-Genome Sequencing (WGS) provided new insights into host-pathogen interactions and the dynamics of disease transmission^11–13^. For example, sequencing the genomes of clinical isolates of *M. bovis* provided an accurate estimates of strain geographical distribution and evolution^14–18^. Such approaches are not common on isolates from Africa and the Middle East, contratry to the analysis of isolates from Euroupe^8^. In this report, we analyzed the genomic diversity and virulence of *M. bovis* isolates from selected dairy herds from the Nile Delta of Egypt. Among examined herds, bTB reached up to 41% on the herd-level but only 3.4% on the animal-level. Both genotyping and WGS indicate the genomic diversity of Egyptian isolates with predominance of isolates being closely related to clonal complex “European 2”. Interestingly, 2 isolates of *M. bovis* were found to be BCG isolates. Further molecular dating analysis allowed us to understand the spatial dynamics and phylogeography of the bTB infection in the Nile Delta, which was further verified by historical events. *In vitro* drug sensitivity testing and mice virulence assays further confirmed strain diversity despite being associated with a specific clonal complex. Overall, we utilized WGS to gain novel insights onto polymorphism and virulence of *M. bovis* isolates circulating in the Nile Delta.

## Results

### Prevalence of bovine tuberculosis in the Nile Delta

**Figure 1:**
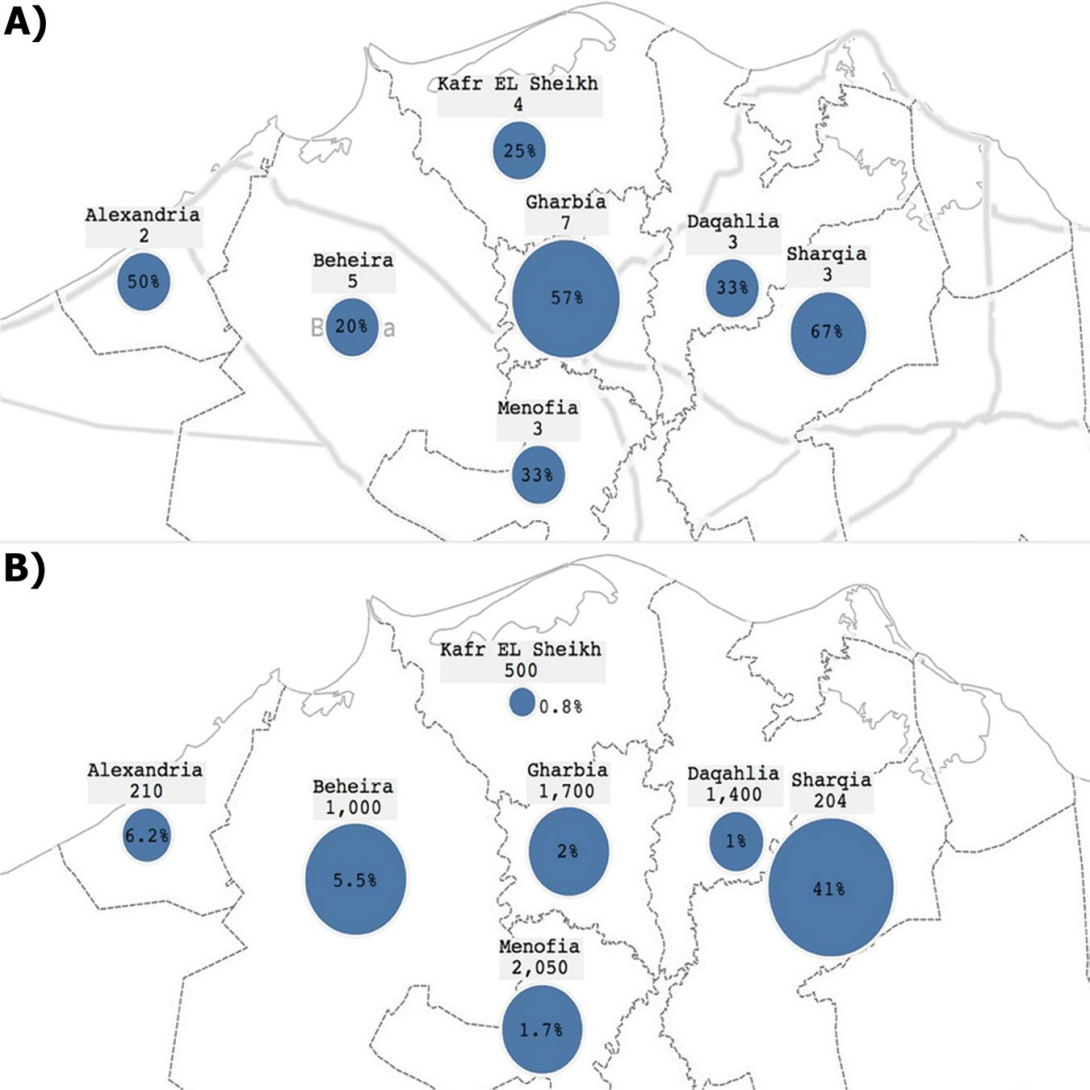
Geographical map of the Nile Delta displaying the prevalence of bovine tuberculosis within several governorates. (**A**) Map showing prevalence of bTB at the herd-level per governorate, (**B**) Map showing prevalence of bTB at the animal-level per governorate. Each governorate shows a number of the total cattle tested and the circle size represents the magnitude of positive results.

Our study design is based on the examination of dairy herds with previous history of bTB followed by the slaughtering of the positive reactors for lesion collection, when possible. The single intradermal comparative skin test (SICST) screening of dairy herds included herds from 7 governorates located in the Nile Delta **(**Fig. 1**)**. A total of 7,064 animals belonging to 27 herds were tested, and only 242 (3.43%) were considered reactors by the applied test standards. However, significant variations in disease prevalence were present within governorates surveyed in this study. For example, the prevelance of bTB was 41% at *Sharqia* but only 0.8% at *Kafr EL Sheikh*, at the animal-level. At the herd-level (presence of at least one positive reactor in the herd), *Gharbia* had the highest number of positive herds (4 out of 7 herds). Only 2 herds were examined from *Alexandria* and only one herd was positive. The overall prevalence of ≥1 positive reactor in herds was 11 among all 27 examined herds (40.74%) **(**Table 1**)**. Finally, the age distribution of the tested animals per governorate showed most to be 3–5 years old, the most productive time in a cow life cycle.

**Table 1:** The number of herds and animals per governorate with age distribution (parenthesis refer to percentages).

Governorate	Herd-level		Animal level				
			Number tested animals	Age distribution			Number positive animals
	Number tested herds	Number positive herds		“<3”	“3–5”	“>5”	
Gharbia	7	4 (57.14)	1700	170 (10.00)	1100 (64.71)	430 (25.29)	35 (2.06)
Daqahlia	3	1 (33.33)	1400	70 (5.00)	930 (66.43)	400 (28.57)	16 (1.14)
Menofia	3	1 (33.33)	2050	200 (9.76)	1350 (65.85)	500 (24.39)	35 (1.71)
Kafr EL Sheikh	4	1 (25.00)	500	70 (14.00)	230 (46.00)	200 (40.00)	4 (0.80)
Sharqia	3	2 (66.67)	204	21 (10.29)	151 (74.02)	32 (15.69)	84 (41.18)
Alexandria	2	1 (50.00)	210	11 (5.24)	176 (83.81)	23 (10.95)	13 (6.19)
Beheira	5	1 (20.00)	1000	179 (17.9)	654 (65.4)	167 (16.70)	55 (5.50)
Total	27	11 (40.74)	7064	721 (10.21)	4591 (64.99)	1752 (24.80)	242 (3.43)

### Nature of lesions associated with bovine tuberculosis

**Figure 2:**
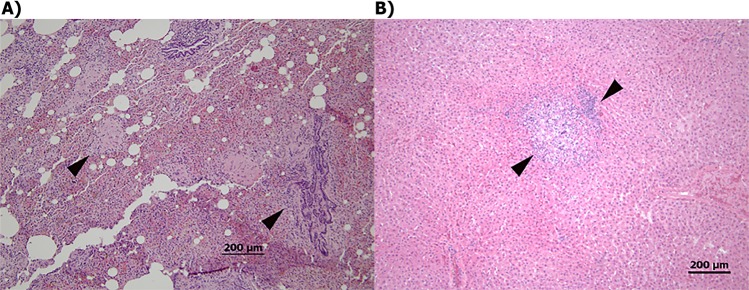
Histology of bovine lungs following post-mortem examination. Tissue sections were taken from bTB positive cases at a participating slaughterhouse. (**A**) Lung with lymphohistiocytic inflammation showing aggregates of macrophages and lymphocytes (arrow heads) and (**B**) Liver with granulomatous inflammatory responses (arrow heads). All sections were stained with H&E and scale bar representing 200 um.

To confirm the initial diagnosis with SICST and to culture isolates from positive reactors, 70 SICST-positive animals were slaughtered and examined by local health authorities. Necropsy lesions from animals are summarized in Table 2. Overall, the highest percentage of the examined tissues showed mixed visible granulomatous lesions (28.57%) in 2 or more systems (e.g. pulmonary and digestive) with the presence of the characteristic lymphocytic infiltration when histology was performed (Fig. 2). Animals with only pulmonary or only digestive lesions also occupied significant proportions of reactors with 25% and 14%, respectively. On the other hand, generalized infections (involvement of multiple systems) were less prevalent (4.29%). Interestingly, *M. bovis* was cultured from 44.29% of the slaughtered animals, even when lesions were not visible **(**Table 2). As expected, primary isolates of *M. bovis* were cultured mainly from mixed and pulmonary lesions, while most of the mycobacterium-negative cultures (N = 15) were from cases with non-visible lesions (NVL, N = 19). Colony morphology, biochemical testing, acid-fast staining and PCR genotyping were used to distinguish *M. bovis* primary isolates (N = 31) from other unclassified slow-growing mycobacteria. Finally, only 11 *M. bovis* isolates were sub-cultured successfully after original isolation (designated MBE for *M. bovis* from Egypt) and were subjected for drug susceptibility testing (DST). Interestingly, 2 isolates (MBE4 and MBE12) were resistant to Isoniazid (INH) while the rest of isolates were fully sensitive to all tested drugs Isoniazid (INH), Rifampicin (RIF), Ethambutol (EMB) and Streptomycin (SM).

**Table 2:** Types and numbers of lesions and culturing results of animals underwent post-mortem examination in slaughterhouses (parenthesis refers to percentages).

Site of infection	Types of Isolated Mycobacteria			Total
	*M. bovis*	Unidentified Slow Grower AFB*	Negative culture	
Pulmonary	10 (55.56)	0 (0.00)	8 (44.44)	18 (25.71)
Digestive	4 (40.00)	1 (10.00)	5 (50.00)	10 (14.29)
Mixed	12 (60.00)	0 (0.00)	8 (40.00)	20 (28.57)
Generalized	3 (100)	0 (0.00)	0 (0.00)	3 (4.29)
NVL*	2 (10.53)	2 (10.53)	15 (78.95)	19 (27.14)
Total	31 (44.29)	3 (4.29)	36 (51.43)	70

*Non-visible lesions indicative of bovine tuberculosis, *Acid-Fast Bacilli.

### Molecular characteristics of *M. bovis* isolates

To gain more insights onto the epizootiology of bTB in the Nile Delta, DNA was extracted from all MBE isolates and subjected to various genotyping protocols. First, restriction fragment length polymorphism (RFLP) based on the *gyrB* gene confirmed that all isolates belong *M. tuberculosis* complex and could be either *M. bovis* or *M. bovis*–BCG. Subsequently, we used PCR to detect the presence of the region of deletion-1 (RD1), identified previously in the genome of *M. tuberculosis* and *M. bovis*^19,20^. Interestingly, all the MBE isolates were virulent *M. bovis* strains except MBE9 and MBE10 which belong to the *M. bovis* BCG species with the characteristic 196 bp band^21^.

**Figure 3:**
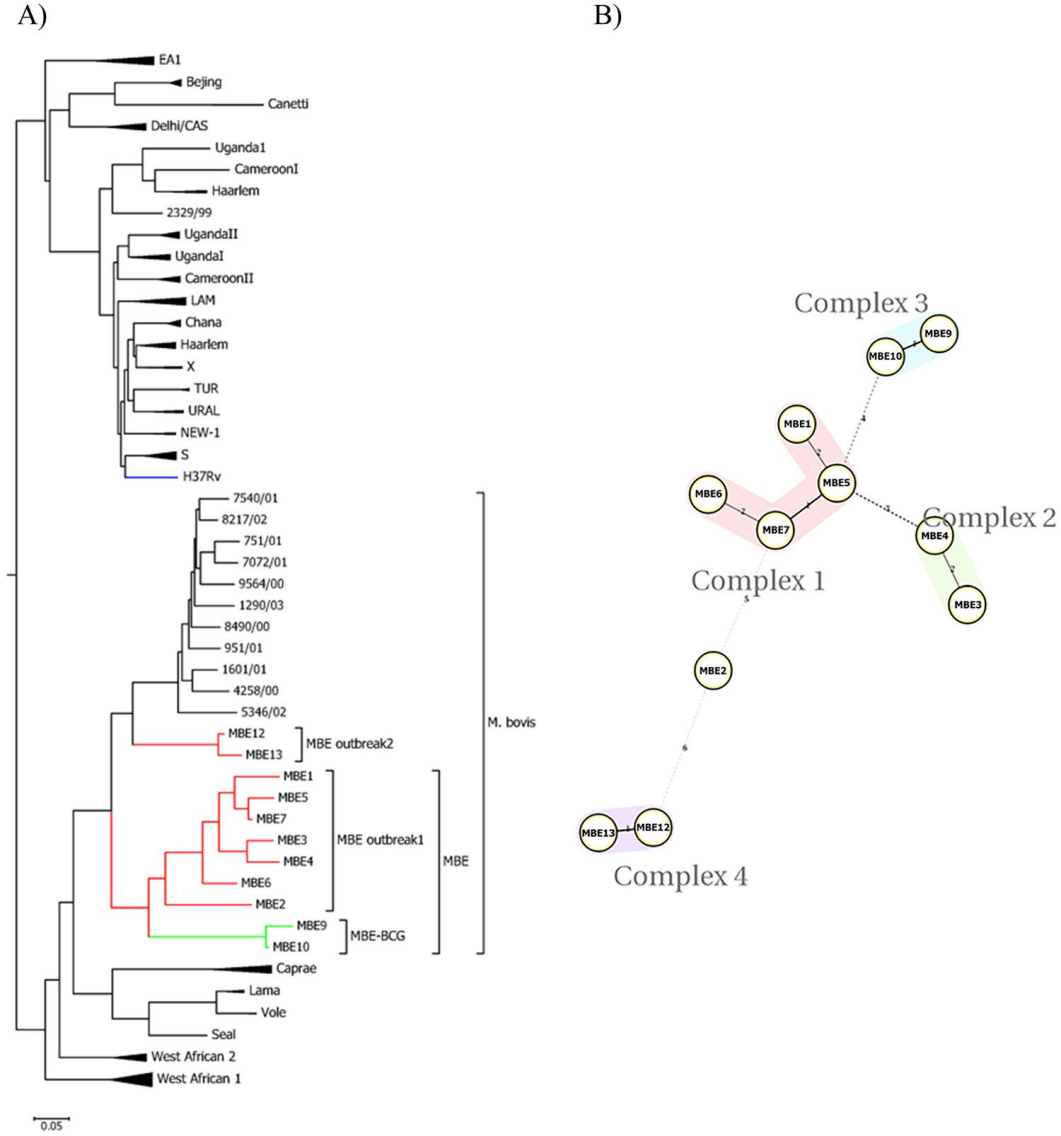
Phylogenetic tree of the clinical *M. bovis* isolates based on the MIRU-VNTR. (**A**) A dendogram was generated using the neighbor-joining algorithm using tools available from the MIRU-VNTR*plus* identification database, (**B**) Minimum spanning tree of 11 *M. bovis* isolates based on the MIRU-VNTR spoligotyping results, categorized based on the identified complexes. Each complex is defined by its number and color code.

Twelve-locus Mycobacterial Interspersed Repetitive Units-Variable Number of Tandem Repeats (MIRU-VNTR) analysis^22^ was perfomed on all MBE isolates to further delineate relationships between isolates from the Nile Delta and those circulating worldwide. As expected, all MBE isolates were clustered with the virulent *M. bovis* except MBE 9 and 10 (BCG cluster) which were closely related but not in the same cluster (Fig. 3A). Interestingly, two isolates (MBE12 and MBE13) which were collected in a more recent outbreak (2 years after the first outbreak) in the Nile Delta showed a distinct but close cluster to the main cluster of MBE Outbreak 1, a clear indication of continous evolution and clonal exapansion of *M. bovis* within the Nile Delta. Further analysis of MIRU-VNTR profiles with minimum spaning tree (MST) algorithm for network analysis^23^, suggested that MBE isolates could be classified into 4 main complexes (Fig. 3B); with pairs of MBE12/MBE13 and MBE3/MBE4 occupying their own complex with a significant genetic distance from the rest of other complexes. Only the MBE2 isolate did not belong to any of the other complexes when MST was used.

### Diversity of *M. bovis* isolates

**Figure 4:**
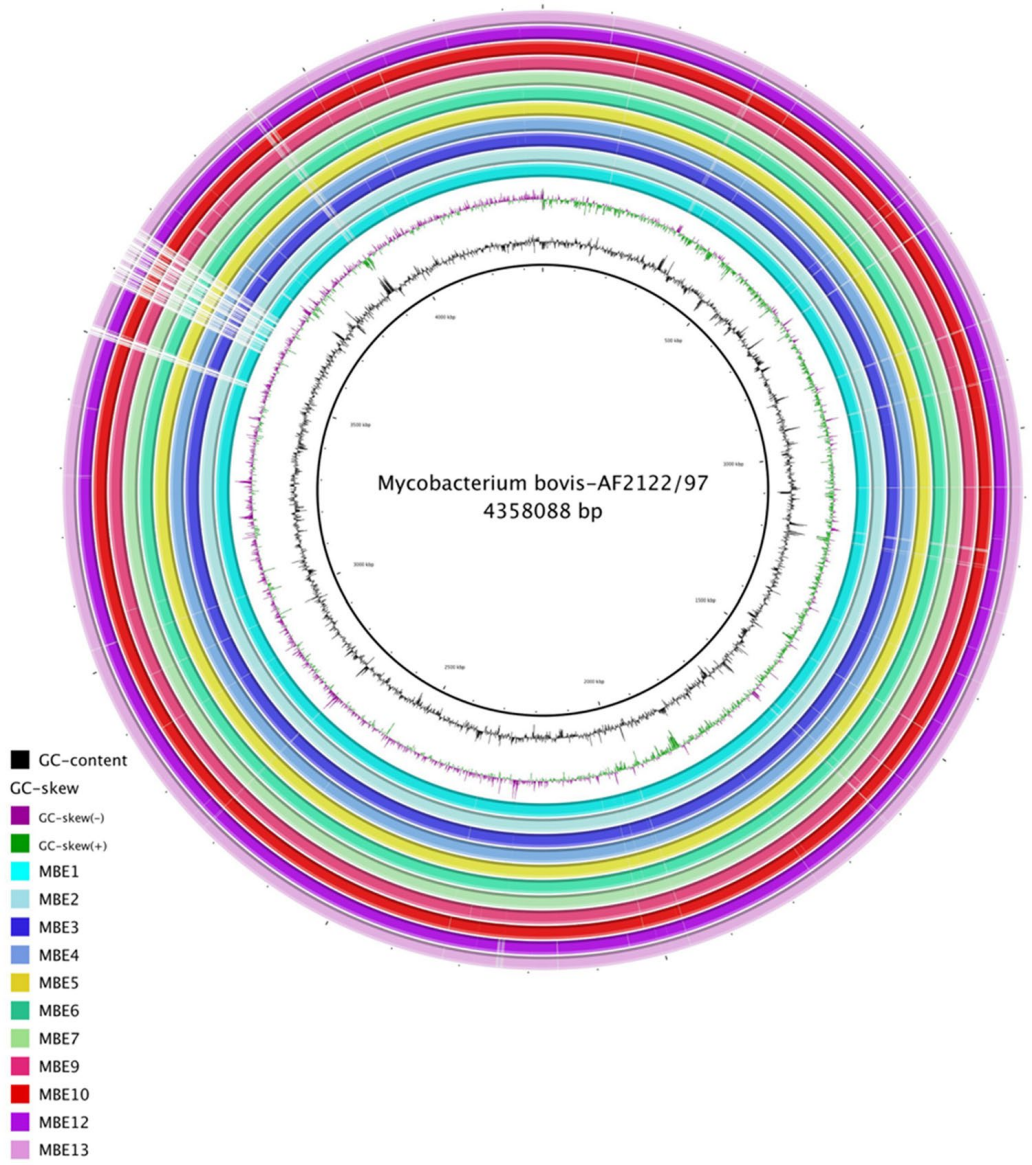
Genomic organization of *M. bovis* from isolates from the Nile Delta. Circular representation showing (from inner to outer), % G + C, GC skew and the homology based on BLASTn+ analysis of the 11 MBE genomes sequentially aligned to the *M. bovis* reference genome strain AF2122/97 (check color legend). The figure was generated using BRIG 0.95. Gene mapping was done using BLASTn with an E-value cut-off 1e-5.

**Figure 5:**
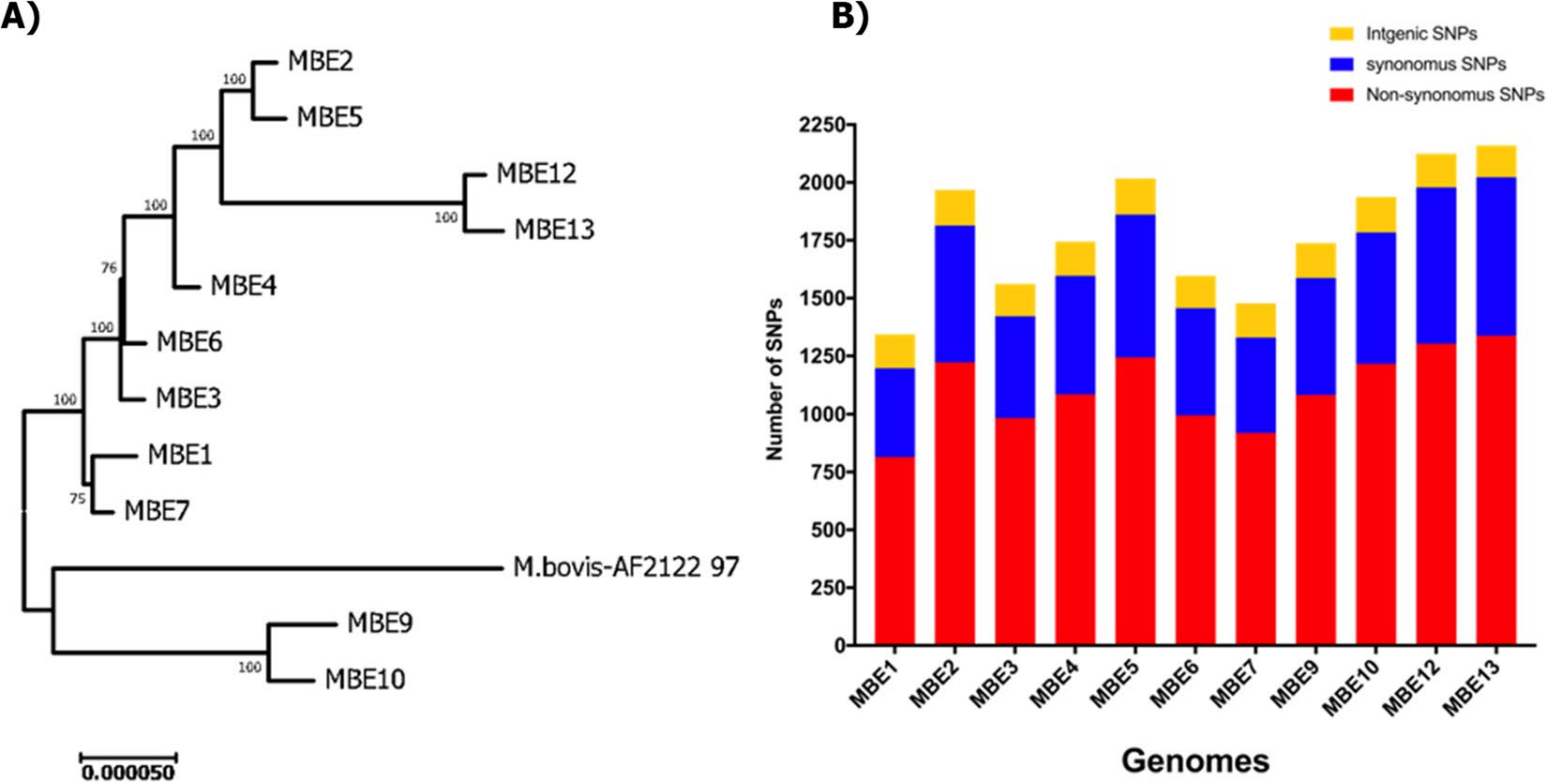
Whole genome sequence analysis of SNPs of *M. bovis* isolates. (**A**) A phylogenetic tree inferred using the neighbor-joining method based on the predicted SNPs of each isolate compared with the *M. bovis* AF2122/97 reference genome. Numbers at each branch represent bootstrap values and branches correspond to partitions with >50% bootstrap replicates. The tree is drawn to a scale with evolutionary distances computed using the maximum composite likelihood method in MEGA7. (**B**) Histogram of the number of SNPs (synonymous, non-synonymous and intergenic) for each examined *M. bovis* isolate compared to the reference genome.

To better characterize variations among MBE isolates on the single nucleotide level, we used Next-generation sequencing technology (Illumina, MiSeq 2000) to generate draft sequences of the MBE isolates. An average of 7,043,390 reads per genome were mapped against the reference genome *M. bovis* AF2122/97 with almost 97.5% reads mapped to the reference genome. Overall sequence analysis indicated that all MBE isolates had genomes which were sequenced on an average of 269 times and an average G + C content of 65.52% representing 97.1% of a genome size estimated to be around 4,231,045 bp (Fig. 4). The calculated average nucleotide identity (ANI) of all the MBE isolates to the reference genome were >99.95 where most of genomes were compiled into ~ 40 contigs with DeNovo assembly. More comparative statistical features of the MBE genomes relative to the reference genome *M. bovis* AF2122/97 are detailed in **(**Table 3). The number of predicted single nucleotide polymorphism (SNPs) for the MBE consensus sequences is shown in **(**Fig. 5**)**. Interestingly, the majority of SNPs (>50%) are non-synonymous (nSNP). Specifically, when 137 virulence related genes were selected for dN/dS analysis, all ratios were >1.0 (Table 3), an indication of a positive selective pressure for virulence genes encoded within *M. bovis* isolates analyzed in this study. The number of predicted SNPs for the MBE2, MBE5, MBE12 and MBE13 consensus sequences was higher than that predicted for the other MBE sequences when aligned to the standard reference genome *M. bovis* AF2122/97. On the other hand, phylogenetic analysis of SNPs from all the isolates confirmed the divergence of MBE10 and MBE9 strains (BCG-like) compared to virulent isolates of *M. bovis* including the distinctive sub-cluster of MBE12 and MBE13 **(**Fig. 5**)**. However, whole genome alignments with *progressive*MUAVE^24^ did not identify major genomic rearrangements among MBE isolates, as expected from members of the *M. tuberculosis* complex due to their clonal nature. Approximately 2–4 locally collinear blocks (LCBs) were found among all MBE genomes relative to the homologous reference genome **(**Supplemental Fig. 2). Overall, all MBE field isolates clustered with *M. bovis* AN5 originally a Brazilian strain used to produce PPD worldwide^25,26^ while the BCG-Like isolates clustered with Russian BCG strain which included the deletion of the RD1 region^19^ (Supplemental Fig. 1).

**Table 3:** Summary of next generation sequencing results.

Parameters	MBE1	MBE2	MBE3	MBE4	MBE5	MBE6	MBE7	MBE9	MBE10	MBE12	MBE13
Year of isolation	2014	2014	2014	2014	2014	2014	2014	2014	2014	2015	2015
Governorate	Gharbia	Gharbia	Beheira	Menofia	Gharbia	Beheira	Gharbia	Alexandria	Alexandria	Menofia	Menofia
Tissue of isolation	Lung LN	Lung Tissue	Pre-scapular LN	Head LN	Generalized	Retropharyngeal LN	Mesenteric LN	Retro-pharyngeal LN	Mesenteric LN	Mesenteric LN	Mesenteric LN
Breed	Cross breed	Cross breed	Holstein	Holstein	Holstein	Holstein	Holstein	Native	Holstein	Native	Native
Age in years	3	5	3–5	5	3–5	>5	5	>5	1–3	>5	>5
Consensus length (Mbp)	4.22	4.24	4.23	4.23	4.25	4.23	4.23	4.23	4.23	4.22	4.22
% of bases	97.10	97.37	97.02	97.55	97.51	97.31	97.39	96.99	97.31	96.59	96.01
% G + C	65.48	65.55	65.52	65.52	65.55	65.51	65.49	65.51	65.53	65.55	65.55
# of reads	2652522	9724874	2190220	2546898	7073852	2381816	2412476	3250964	3342700	22446678	19454286
Average Coverage	140.00	514.35	115.36	135.38	374.50	126.49	127.89	170.80	175.53	584.96	498.29
% of mapped reads	95.91	96.75	96.14	97.16	97.08	96.53	96.69	95.77	96.72	96.41	95.8
Mean mapped read length	241.59	240.7	240.41	240.56	239.94	241.3	240.27	241.43	238.4	120.67	119.91
Mean paired read distance	439.76	418.69	443.67	429.86	451.43	445.05	453.91	407.74	417.31	172.10	173.46
# of contigs	90	58	83	73	61	75	83	81	71	85	83
N50	104531	150169	119829	119870	155216	117225	117190	119771	118077	107878	115326
ANI to *M. bovis* AF2122/97	99.96	99.95	99.96	99.96	99.96	99.97	99.95	99.95	99.96	99.95	99.95
# of InDels against M. bovis Af2122/97	53	61	53	44	68	51	54	30	27	286	343
# of SNPs against *M. bovis* Af2122/97	1343	1968	1561	1744	2017	1597	1477	1738	1937	2124	2160
Non-synonymous SNP	815	1222	984	1084	1244	995	919	1082	1216	1303	1338
Synonymous SNP	382	592	438	513	617	462	411	505	568	676	685
Intergenic SNP	146	154	139	147	156	140	147	151	153	145	137
dN/dS*	1.8059	1.7800	1.7220	1.8060	1.7798	1.7363	1.7086	1.9140	1.9483	1.6203	1.8364
*In-silico* Spoligotyping	SB0268	SB0268	SB0268	SB0268	SB0268	SB0268	SB0268	SB0120	SB0120	unreported	unreported

*Calculations of dN/dS ratio is based on selected 137 virulence related genes listed in Supplemental Table 1.

To better trace the origin of MBE isolates within the Nile Delta, we used the SpoTyping tool that utilizes WGS to identify the characteristic spoligotype for each isolate^27^. The majority of MBE isolates fell within the SB0268 (MBE1-MBE7) which is the spoligotype of the Brazilian strain *M. bovis* AN5 and also found in united Kingdom and Mexico^28^. The MBE9 and MBE10 belonged to the SB0120 spoligotype, which is the spoligotype of BCG-like human *M. bovis* isolates previously found in Tunisia and Italy^29,30^. Finally, the MBE12 and MBE13 spoligotypes are of unknown SB number, suggesting a new spoligotype. Interestingly, the *in silico* analysis of the clonal complexes identified that all virulent MBE isolates were missing the spacer 21 plus the presence of the *gua*A SNP, a characteristic for the Clonal Complex European 2^31^. This clonal complex is known to be prevalent in Brazil^8,31^, Portugal, Spain, and at a low frequency in both France and Italy^31^.

### Dynamics of bovine tuberculosis transmission in the Nile Delta

**Figure 6:**
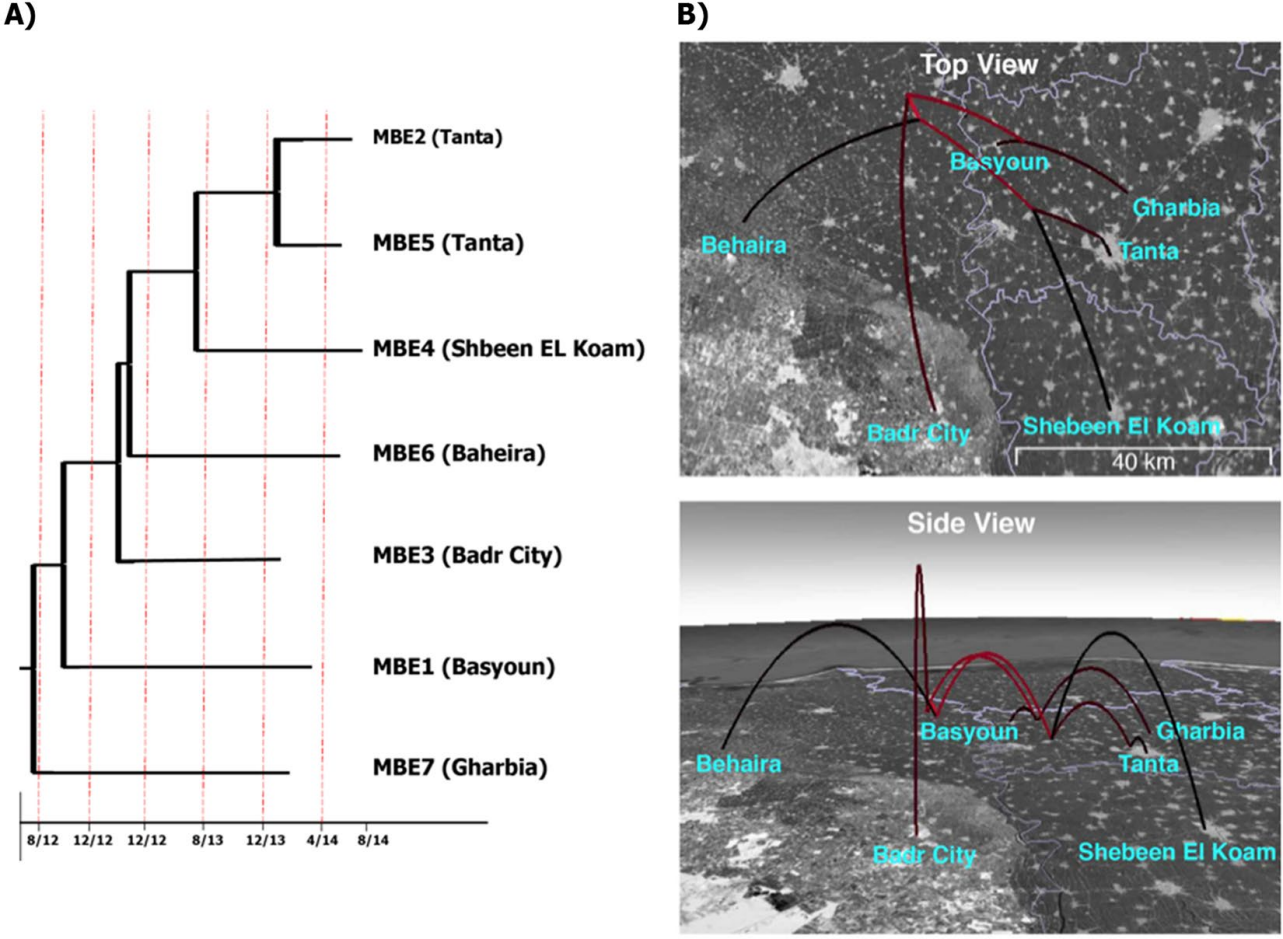
Divergence times and phylogeographic model of bovine tuberculosis in the Nile Delta. The examined outbreak originated from a single common ancestor in early 2012, and rapidly spread among the sampled localities. (**A**) Divergence time estimates. Tip names indicate the isolate name and locality. (**B**) Phylogeographic model of the studied outbreak of bovine tuberculosis. The phylogeny from A is projected onto a map of the Nile Delta region of Egypt. Red colored branches represent more ancestral branches of the phylogeny. Maps were created by SPREAD V.1.0.7^80^.

To better evaluate the dynamics of bTB transmission within the Nile Delta, we traced both herd historical records and molecular dating records of virulent isolates of *M. bovis* from Egypt. Our initial analysis incorporating all MBE isolates confirmed the monophyly of the non-BCG isolates from Egypt (Supplemental Fig. 3). Molecular timing analysis estimated that those isolates have originated about 3–4 years prior to the most recent sampled isolate. Subsequent phylogeographic analysis indicated a single independent transmission into each of the localities from which isolates were derived (Fig. 6). For example, the two isolates from *Tanta* are each other’s closest relatives, supporting a scenario where bTB was spread among individuals within the same region. While we are unable to identify the original source of the infection in the region, the phylogeographic model suggests transmission among these localities all within a matter of months during this particular outbreak. Dates of first case diagnosis and the start of the local bTB outbreak confirmed the scenario proposed by molecular dating analysis.

### Virulence of *M. bovis* isolates

**Figure 7:**
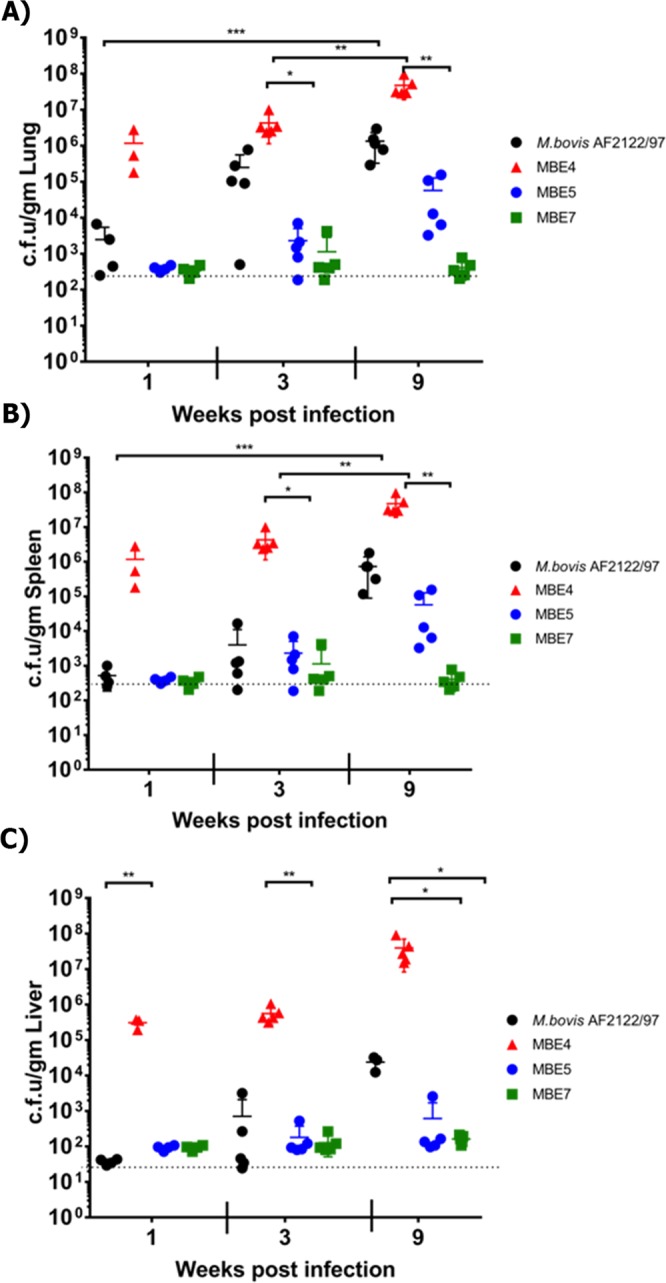
Tissue colonization of MBE isolates. Groups of BALB/c mice were infected by aerosol route with either MBE4, MBE5 and MBE7 in comparison with the reference *M. bovis* strain AF2122/97. Lungs, spleens and livers from *infected* animals were harvested and cultured at different weeks post infection. Each circle represents the colonization level for each organ from one animal. Asterisks (* for *p* < *0.05* and ** for *p* < 0.005) indicate statistical significant difference in colonization level between each clinical isolate compared to the reference strain.

**Figure 8:**
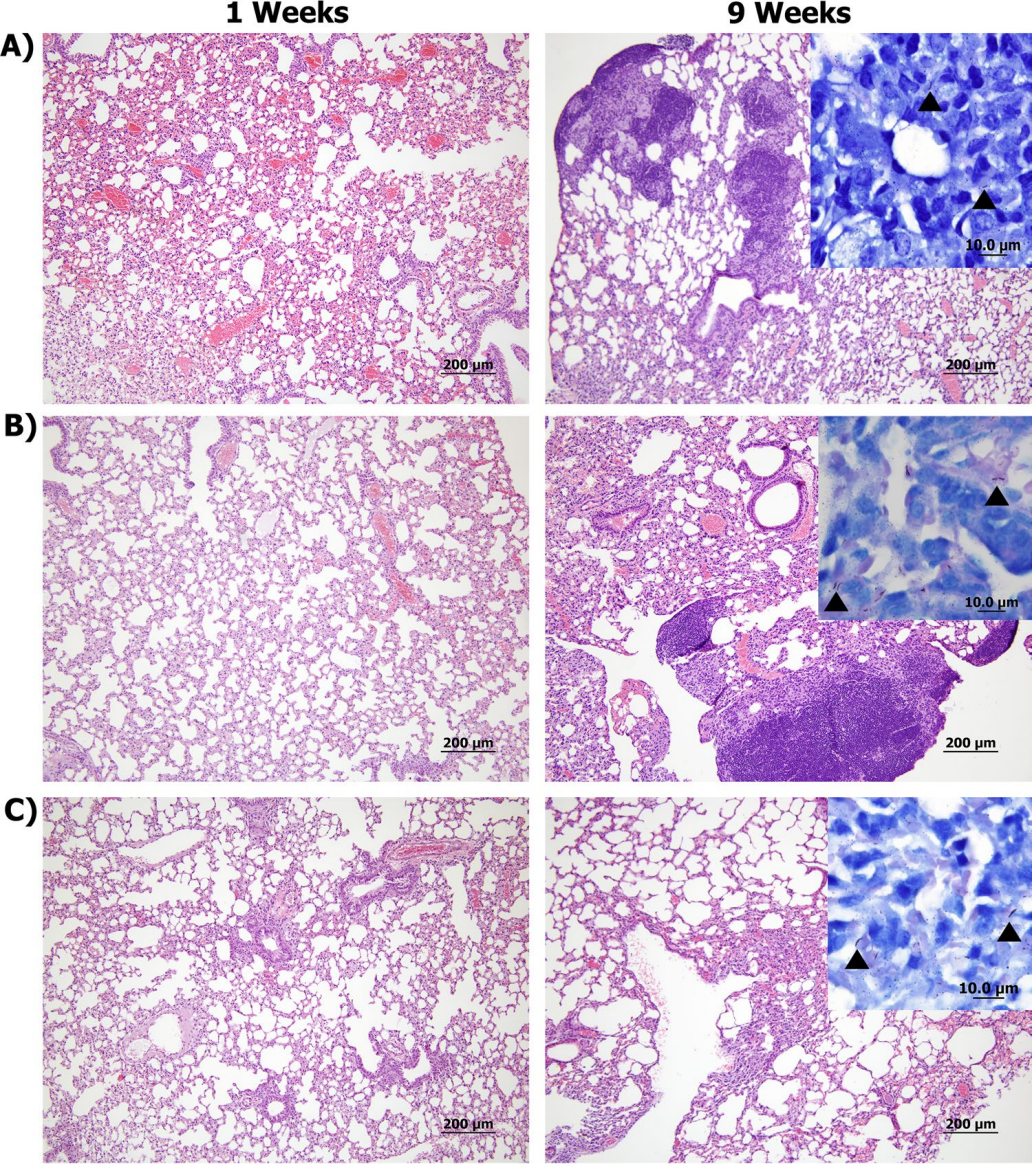
Histopathology of mice infected with clinical and reference isolates of *M. bovis*. Tissues sections stained with H&E collected from mice lungs infected with *M. bovis* AF2122/97 (**A**), MBE4 (**B**), and MBE7 (**C**) at 1 (left) and 9 (right) weeks post infection are shown at 40 × magnification (scale bar = 200 μm). Insets showing Ziehl-Neelsen-stained lung sections are also included, with arrowheads indicating acid-fast bacilli at 1000× magnification (scale bar = 10 μm). No bTB-associated granuloma infiltrates or acid-fast bacilli were found in any tissues in the naive group. Only tissue sections from mice infected with MBE7 were shown because of histopathology similarity between both MBE5 and MBE7 infections.

To evaluate potential differences in virulence among examined MBE isolates, mycobacterial colonization levels and histological analysis of murine tissues were analyzed. The selected isolates (MBE4, MBE5, and MBE7) were representing different geographical locations with disinctive genotypes (based on phylogenetic and molecular timing analysis). Mouse groups were sampled at 1, 3, and 9 weeks post-infection to represent early, middle, and progressive stages of infection following aerosol infection. As expected, low bacterial burden was found in all examined tissues at 1-week post-infection (WPI) with MBE5 and MBE7 **(**Fig. 7**)**, suggesting decreased ability to initiate infection compared to MBE4. This low-colonization profile for MBE5 and MBE7 continued at 3 and 9 WPI. For the standard *M. bovis* isolate (AF2122/97), colonization levels increased at 3 WPI but peaked by 9 WPI, as expected. Histologically, mild inflammatory responses in murine lungs, spleen and liver were noticeable (score 1–2) by 3 WPI with the reference *M. bovis* strain AF2122/97. However, as infection progressed, the inflammatory responses were intensified in lungs by 9 WPI (score 3) with the reference strain (Fig. 8). Interestingly, lesions from mice infected with MBE4 were first observed in spleen by 3 WPI (score 2) but then more noticeable in the lungs by 9 WPI (score 2–3). Such unique histological profile could suggest a tendency of MBE4 to establish systemic infection by early infection of spleen, similar to the standard virulent strain. Unlike MBE4, all other tested clinical isolates (MBE5, MBE7) did not induce measurable levels of inflammation throughout the 9 WPI (score 0–1), consistent with their low levels of bacterial colonization.

To further analyze the difference among MBE isolates; a detailed analysis of SNPs and insertions/deletions regions (InDels) was done to profile potential correlates with increased virulence. Several SNPs were identified for MBE4 including the absence of specific SNPs from MBE4 while those SNPs were present in the rest of the isolates. Interestingly, 5 SNPs were present only in the PE_PGRS14 of MBE5 and MBE7 isolates, all were confirmed by Sanger sequencing. The PE_PGRS14 gene is a member of the *M. tuberculosis* complex PE family, PGRS subfamily of glycine-rich proteins involved in virulence^32^, as shown from its upregulation during acute phase of macrophage infection^33,34^. In addition, MBE4 had the least number of InDels (n = 34) from the reference virulent strain when compared to MBE5 (n = 53) and MBE7 (n = 37). Among the InDels of MBE4, a fragment of 106 bp length (Supplemental Table 4) was deleted only from PGRS14 genes but present in the rest of isolates. It is noteworthy to mention here that several SNPs and InDels (Supplemental Tables 3 and 4) were present throughout the genomes of MBE4, MBE5 and MBE7 but not associated with PE-PGRS14 with potential impact on strain virulence. For example, a unique insertion was found in MBE4 associated with moeB1 (BQ2027_MB3231C), a probable molybdenum cofactor biosynthesis protein with potential impact on virulence^35^.

## Discussion

In Africa and other developing countries, *M. bovis* accounts for approximately 10–20% of cases of human tuberculosis^36^. The global prevalence of human *M. bovis* infection is higher among patients with extra-pulmonary tuberculosis, since the pathogen is frequently acquired via oral ingestion and gastrointestinal disease is an important clinical manifestation^37^. Understanding disease transmission dynamics of *M. bovis* in cattle could eliminate such infections in humans. In Egypt, a previous investigation of health centers indicated presence of *M. bovis* in 0.4% to 6.4% of sputum-positive samples^38^. In this report, we examined the status of bTB infection in the Nile Delta of Egypt on both ecological and genomic levels. Our investigation traced cases of bTB in dairy herds of 7 governorates with a previous history of *M. bovis* infection. Historically, the General Organization of Veterinary Services (GOVS) indicated that bTB in slaughtered cattle in the Egyptian abattoirs was 0.05% in 1989. In subsequent years, the percentage of positive reactors in some governorates reached 11–23%^39^. In the present study, the SICST test indicated that 40.74% of the herds were tested positive for *M. bovis*-infection. At the animal-level, only 3.25% of animals were SICST-positive, similar to earlier reports^6,7^. Bovine tuberculosis may be kept under control by a national campaign of continuous testing and slaughtering of reactors, but the impact of emerging new virulent isolates remains to be assessed. As expected, animals >3 years-old were more often SICST-positive compared to younger animals. This may be due to the long incubation period and stress of high milk production in this age class^40^. Interestingly, 27% of SICST reactors had no visible bTB lesions, which was significantly higher than earlier studies^41,42^. This high level of potential false-positive SICST reactors could be attributed to infection with mycobacteria other than *M. bovis* or poor sensitivity of the postmortem examination^43^. More importantly, pulmonary and mixed type lesions were the most often observed lesions, suggesting the respiratory route to be the most important route of infection in the studied herds.

A surprising finding in our study was the isolation of BCG isolates from suspected cases of bTB in the Nile Delta. To our knowledge, previous isolation of BCG-like isolates from animals accounted for 26% of isolates in one study based on spoligotyping without any further confirmation to these isolates identity^44^, however, isolation of *M. bovis* BCG itself from animals is expected to be rare. In fact, BCG isolates were noticed in human infections with *M. bovis*^29^, mainly with the same spoligotype of the BCG isolates described in this study (SB0120). Other than illegal vaccination of animals with *M. bovis* BCG, this finding might imply a potential transmission from immune-compromised individual vaccinated earlier with BCG. More focused analysis of the virulent MBE isolates indicated their association with SB0268 spoligotype, suggesting limited genetic variations among isolates of *M. bovis* circulating in Egypt. Nevertheless, MIRU-VNTR indicated the presence of 4 complexes with more diversity than discovered by spoligotyping. Such analysis provided enough justification for WGS to uncover further information that could not be detected by analysis of limited sequence targets.

As expected, WGS showed some subtle differences amongst the 11 MBE genomes. Comparative genome analysis of the MBE genomes against a standard *M. bovis* reference (AF2122/97) identified additional genome-wide features that could not be deduced from specific gene analyses, as it provided higher levels of analysis at a single nucleotide level (SNPs). Further analysis of other SNPs indicated that recent isolates (MBE12 and MBE13) had a higher number of SNPs and constitute a separate clade of isolates from those belong to the first outbreak (MBE1-MBE10), another indication of the emergence of new foci of infection with their own potential virulence characteristics. Interestingly, one of the isolates (MBE4) has a unique genotype compared to other isolates (e.g. MBE5, and MBE7) from the Nile Delta including 5 SNPs and a 106 bp deletion in the PE_RGRS14 gene alone. PE_PGRS14 (Rv0834c) is thought to be upregulated in human lung granulomas compared to *in-vitro* grown bacteria^34^. Members of the PGRS subfamily of PE proteins are thought to be associated with cell wall or surface exposed^45^ with a potential role in mycobacterial pathogenesis^46^ and the evasion of host defenses^47^. However, given the wide polymorphism within MBE4 genotype (additional 22 SNPs outside the PGRS14 gene), it is difficult to establish virulence association without further studies. The fact that spleens from MBE4-infected mice showed inflammatory responses during the first 3 weeks of infection could suggest a highly successful isolate for establishing systemic infection during early stages of the disease. Earlier reports suggested that early dissemination of *M. tuberculosis* to the spleen, is usually associated with increased immune responses to the infection^48^, as evident from our histopathology analysis. Analysis of further time points with targeted gene deletions could reveal the potential virulence for MBE5 and MBE7 isolates, a logical extension of this project.

Overall, we were able to use all SNPs identified in all isolates to predict the dynamics of *M. bovis* isolates within the Nile Delta using a molecular timing algorithm. This approach is very helpful to show how *M. bovis* was able to transfer among dairy herds within a relatively short period of time (3–4 years), another reason to establish better control programs for bovine tuberculosis. Moreover, identified SNPs indicated the potential origin of recent outbreak of bTB in the Nile Delta from isolates present in Latin America and several European countries. The apparent lack of isolate genotypes from African countries with documented animal trade with Egypt (e.g. Ethiopia) and a known history of enzootic bTB^49,50^ was to some extent, surprising. It is possible that isolates from Africa are not well-represented in the *M. bovis* databases or historical trades with European countries allowed for successful spread within the Nile Delta. Analysis of more isolates from African countries, including Egypt, could further improve our understanding of the nature of *M. bovis* transmission and virulence.

## Materials and Methods

### Identifying naturally infected animals with bovine tuberculosis

The present study was based on the screening of herds with known history of bTB between 2013 to 2015, conducted by the Veterinary Serum and Vaccine Research Institute (VSVRI) and General Organization of Veterinary Services (GOVS) in Egypt. A total of 27 dairy herds from 7 governorates **(**Fig. 1**)** were included in this study based on previous reporting of suspected cases of bTB. The initial herd testing included whole-herd screening using Single Intradermal Comparative Skin Tuberculin (SICST) test administered in one side of the neck according to the OIE manual for diagnostic tests and vaccines for terrestrial animals^51^. Positive reactors (≥4 mm skin induration in bovine tuberculin injection site compared to avian tuberculin injection site) were subjected to culling then postmortem examination to inspect lungs, liver, kidneys, udder and regional lymph nodes. All tissue specimens were carefully examined for any gross lesions associated with bTB such as granuloma formation or caseation and calcification of parenchymal lymph nodes^52,53^.

### Bacterial isolation and genomic DNA extraction

All organs and tissues showing gross lesions were collected. Samples were decontaminated by Hexadecyl-Pyridinium Chloride (HPC) as previously described^54,55^. Briefly, HPC was added to the homogenized tissues at room temperature for 15 min, tissue suspensions were centrifuged for 15 min at 1000Xg and the obtained sediments were inoculated into 2 glycerinated and 2 pyruvate Lowenstein-Jenson slants. All slants were incubated at 37 °C in inclined position for overnight, then vertically for at least 6–8 weeks. The obtained colonies were observed for morphological character and for pigment production^56^. A total of 31 *M. bovis* isolates were obtained from clinical samples taken from suspected tuberculous lesions from animals that scored positive in the intradermal tuberculin test, only 11 isolates were successfully sub-cultured. All the isolates (designated MBE for *M. bovis* Egypt) were grown in 10 ml of Middlebrook 7H9 liquid media supplemented with 0.36% sodium pyruvate and 10% ADC (Albumin-Dextrose-Catalase) for 4–5 weeks using BSL3 practices according to our approved biosafety protocol from the University of Wisconsin-Madison. High-quality genomic DNA (gDNA) samples were isolated as detailed before^57^ and its quality verified by both NanoDrop (Thermo Scientific, Wilmington, DE) and gel electrophoresis. In addition to clinical isolates, DNA samples from cultures of *M. bovis* AF2122/97, *M. bovis*-BCG (Pasteur) and *M. tuberculosis* H37Rv were isolated to serve as controls. All cultures of *M. bovis* isolates were handled under BSL3 environment at the University of Wisconsin-Madison.

### Drug susceptibility testing

All clinical isolates were subjected to a standard drug susceptibility test against 4 first-line drugs isoniazid (INH), rifampicin (RIF), ethambutol (EMB) and streptomycin (SM) using the disk elusion assay (DEA)^58^. The following drug concentrations were used, INH, 0.2 μg/mL; RIF, 5.0 μg/mL; SM, 10.0 μg/mL; EMB, 5.0 μg/mL, according to Clinical and Laboratory Standards Institute (CLSI) guidelines M24-A2^58^. Each strain was scored as resistant to a specific drug if its growth rate was >1% compared to the control isolate growth.

### Molecular typing

The MBE isolates were genotyped using the PCR-RFLP of *gyrB* gene for general species identification among members of *M. tuberculosis* complex as described before^59,60^. In the first step, a 1020 bp fragment of the *gyr*B gene was amplified with the specific primers MTUB^60^. In the second step, amplicons were digested with restriction enzymes *Rsa*I, *Taq*I, and *Sac*II and the generated band pattern was examined to match members of the *M. tuberculosis* complex. To differentiate between members of the *M. bovis* cluster (virulent *M. bovis* and vaccine *M. bovis*-BCG), the RD1 typing were done utilizing RD1-F: AAGCGGTTGCCGCCGACCGACC, RD1-R: CTGGCTATATTCCTGGGCCCGG, and RD-1-R2: GAGGCGATCTGGCGGTTTGGGG primers^61^. Additionally, all MBE isolates were analyzed using the Mycobacterial Interspersed Repetitive Units-Variable Number of Tandem Repeats (MIRU-VNTR) method^22,62–64^. A panel of 12 MIRU loci was targeted for MIRU-VNTR genotyping^62^ and results were analyzed using a MIRU-VNTR*plus* database^65^.

### Whole genome sequencing and comparative genomic analysis

Next-generation sequencing using the Illumina-MiSeq 2000 platform was performed at the University of Wisconsin-Madison Biotechnology Center as detailed before^66^. Raw sequence reads with average read length of 250 bp, were assembled against the sequence of the reference strain *M. bovis* Af2122/97^67^ using CLC-Bio Genomic Workbench version 8.0.1. Single nucleotide polymorphisms (SNPs) and insertion and deletion (InDels) polymorphisms were also identified using algorithms implemented by the CLC Genomics Workbench 8.0.1. However, repetitive genomic regions were not excluded from SNP predictions which could lead to discovery of false SNPs. For SNP predictions, parameters were set as follows: average base quality filter cutoff 15, central base quality filter cutoff 20, minimum sequence coverage 20, minimum variation frequency cutoff 50%, and maximum variation 2. All SNPs predicted from PE_PGRS14 that differed between genomes (N = 5) were further confirmed by Sanger sequencing^68^. For the In-Del analysis, parameters were set as follows: minimum sequence coverage was set to 10 and minimum variation frequency cutoff was 50%. The concatenated SNP files for each strain were aligned with MEGA V7.0.26^69^ and used to build a Neighbor-Joining phylogenetic tree (Fig. 5) with 1000 bootstrap replications and Jukes-Cantor substitution model^70^. The dN/dS ratio was calculated based on the codons of the 137 virulence related genes (Supplemental Table 1) using the Nei and Gojobori (Jukes-Cantor)^71^ and incorporating a statistic developed in Ota and Nei^72^ method using SNAP v2.1.1 implemented in HIV sequence database (https://www.hiv.lanl.gov/content/sequence/SNAP/SNAP.html).

To provide a robust measurement of genetic distance among bacterial genomes, the average nucleotide identity (ANI) was calculated through ANI calculator on EzGenome (http://www.ezbiocloud.net/tools/ani). Multiple whole-genomic alignments were performed using the Harvest suit^73^ allowing the identification of the gene or the intergenic region where differences are located. A phylogenomic tree (Supplemental Fig. 2) was constructed using a total of 67 *M. bovis* genomes retrieved from the NCBI public database for comparative purposes (Supplementary Table 2). The phylogenomic tree is based on the genomic polymorphism found at the core genome sequence shared with a similarity over a threshold between all genomes included in the analysis (Supplementary Fig. 1). The dendogram is visualized with CLC-Bio Genomic Workbench version 8.0.1. To analyze genome-wide rearrangements, sequences of all MBE genomes were aligned against the *M. bovis* AF2122/97 using MAUVE multiple alignment software with the progressive alignment option^24,74,75^ (Supplementary Fig. 2).

For the detection of *in silico* spoligotypes of MBE, we used SpoTyping-v2.1^27^ by analyzing raw sequence reads obtained from Illumina MiSeq 2000 platform. SpoTyping is implemented with the Python language and BLAST algorithm. The binary and octal code representation of the spoligotypes were compared using the *M. bovis* Spoligotype Database (www.mbovis.org). For the identification of *M. bovis* clonal complexes^76^, *In silico* analysis of the 4 *M. bovis* clonal complexes were investigated among the MBE genomes consisted in the detection of RDAf1 and deletion of spacer 30 for African 1 using 3 previously described primers^77^. Detection of RDAf2 and deletion of spacers 3–7 for African 2 using another set of 3 previously described primers^49^. Detection of RDEu1 and deletion of spoligotype spacer 11 for European 1 using a pair of previously described primers^78^. Detection of *guaA* gene SNP at 3,765,573 position according to the reference genome (*M. bovis* AF2122/97) and deletion of spoligotype spacer 21 were used for the identification of European 2 clonal complex^76^.

### Molecular timing

To analyze the patterns of bTB introduction and transmission throughout the Nile Delta, we employed an approach based on molecular clock and phylogeographic analyses. To establish an estimate of the age of the most recent common ancestor (MRCA) of the MBE isolates, we first analyzed the genomic SNP dataset that incorporated previously published bTB genomes from around the world, using BEAST 2.4.7. We used a GTR model of molecular evolution, which was identified as the best fitting model with jModelTest^79^. We placed a lognormal prior on the substitution rate (mean = 0.005 substitutions per site per year, SD = 0.3), and allowed these rates to vary among lineages, following previously estimated rates. We ran two chains of 100 million generations, assessing convergence using Tracer v1.6. We used the ages resulting from this first analysis as the basis for a phylogeographic analysis of a single outbreak of MBE isolates (isolates MBE1–7), which was estimated to be about 3.4 years prior to the most recently collected sample (September 2014). We therefore placed a prior under a normal distribution with an offset of 7 years on the MRCA of these isolates, for which we also indicated the tip dates. We placed a conservative exponential prior on rates of geographical spread and allowed these to vary among branches. Due to the smaller sampling size of this second analysis, we ran two chains of 20 million generations. We used Spread v1.0 to visualize the phylogeographic model^80^ available at https://github.com/phylogeography/SPREAD.

### Virulence determination

Male and female BALB/c mice groups (n = 14) at 5–6 weeks age were infected using the *M. bovis* field isolates MBE4, MBE5, and MBE7 in comparison with the *M. bovis* AF2122/97 reference strain. Approximately 50–100 CFU were administered by aerosol using the Glas-Col inhalation system (Glas-Col, LLC, Terre Haute, IN). The infectious dose for each group was confirmed by plating lungs of an infected mouse at 1-day post-challenge. At 1, 3 and 9 weeks’ post-challenge 4–5 mice were sacrificed from each group for bacteriology and histopathology as detailed before^81^. Briefly lung, spleen, and liver samples were homogenized in PBS, brought up to a total volume of 2 ml before plating undiluted and 10-fold serial dilutions of samples onto 7H10 Middlebrook plates. For histopathology, sections were cut from embedded samples and stained with H&E or Ziehl–Neelsen for acid-fast bacilli. Histological lesions were scored by a trained pathologist blinded to the expperimental groups using a severity scale of 0 to 5 where 1, minimal; 2, mild; 3, moderate; 4, severe; and 5, massive. Images of lungs at 40× magnification were also analyzed in Photoshop CS2 (Adobe, San Jose, CA) to determine the percent of the inflamed area of the lung as compared to the total area of the lung for each animal post challenge. For statistical analysis, One-way ANOVA with Bonferroni’s post-test was used in Prism 5.01 (GraphPad Software). The post-test P values are as follows: *p < 0.05; **p < 0.01; ***p < 0.001.

### Ethics statement

All procedures for animal testing, euthanasia, and sample collection were carried out at the governmental veterinary regional slaughterhouses, General Organization of Veterinary Services (GOVS), Nile Delta, Egypt. Euthanasia of animals was carried out in regional abattoirs in accordance with the recommendations by the Code of Practice for the Care and Handling of Dairy Cattle (http://www.nfacc.ca/codes-of-practice/dairy-cattle). All animal experiments were approved of by the Institutional Animal Care and Use Committee, University of Wisconsin-Madison. All laboratory procedures and techniques described in this report were conducted in accordance with the relevant guidelines and regulation of the University of Wisconsin-Madison.

## Supplementary information

Supplemental material

## Data Availability

The genome sequences and annotated sequence files for all MBE isolates reported here have been deposited in GenBank under the accession numbers (MBE1: QFZD00000000, MBE2: QFZC00000000, MBE3: QFZB00000000, MBE4: QFZA00000000, MBE5: QFYZ00000000, MBE6: QFYY00000000, MBE7: QFYX00000000, MBE9: QFYW00000000, MBE10: QFYV00000000, MBE12: QFYT00000000, MBE13: QFYU00000000).
