# Supplementary material for: Genomic Polymorphism Associated with the Emergence of Virulent Isolates of Mycobacterium bovis in the Nile Delta
Source: Sci Rep. 2019 Aug 12;9:11657. doi: 10.1038/s41598-019-48106-3 (PMC6690966; doi:10.1038/s41598-019-48106-3)
Supplement: Supplementary file 1 — Supplemental material [file 41598_2019_48106_MOESM1_ESM.pdf]

Genomic Polymorphism Associated with the Emergence of Virulent Isolates of *Mycobacterium bovis* in the Nile Delta.

Hazem F M Abdelaal[1], Daniel Spalink[2], Ali Amer[3], Howard Steinberg[1], Emad A Hashish[4], Essam A Nasr[5], Adel M Talaat[1*]

[1]Department of Pathobiological Sciences, University of Wisconsin-Madison, Madison, WI, USA.

[2]Department of Ecosystem Science and Management, Texas A&M University, College Station, TX, USA.

[3]Animal Health Research Institute, Dokki, Giza, Egypt.

[4]Department of Clinical Pathology, Faculty of Veterinary Medicine, Zagazig University, Zagazig, Egypt.

[5]Veterinary Serum and Vaccine Research Institute, Bacterial Diagnostics Research Department (Tuberculosis), Abbasia, Cairo, Egypt.

Running Head: Molecular Diversity of *M. bovis* Isolates from Egypt

#Address correspondence to:
Adel M. Talaat, M.V.Sc., Ph.D.
Lab of Bacterial Genomics
Department of Pathobiological Sciences
School of Veterinary Medicine
University of Wisconsin-Madison
Robert P Hanson Biomedical Science Laboratories
1656 Linden Drive, Madison, WI 53706-1581
Tel: 608-262-2861
Fax: 608-262-7420
adel.talaat@wisc.edu

Supplemental Table 1. List of 137 virulence related genes[1] predicted in the *Mycobacterium bovis* reference genome (*M. bovis AF2122/97*) used for the calculation of *dN/dS* ratios in the *M. bovis* isolates from the Nile Delta.

#	Gene Name	Rv number	Mb number
1	treS	Rv0126	Mb0131
2	ptbB	Rv0153c	Mb0158c
3	mce1r	Rv0165	Mb0170c
4	fadD5	Rv0166c	Mb0172c
5	sigG	Rv0182c	Mb0188c
6	zmp1	Rv0198c	Mb0204c
7	pckA	Rv0211	Mb0217
8	hsp	Rv0251c	Mb0257c
9	mosR	Rv0348	Mb0356
10	hspR	Rv0353	Mb0361
11	pknG	Rv0410c	Mb0418c
12	sodC	Rv0432	Mb0440
13	icl1	Rv0467	Mb0467
14	pcaA	Rv0470c	Mb0479c
15	hbhA	Rv0475	Mb0485
16	-	Rv0485	Mb0495
17	senX3	Rv0490	Mb0500
18	regX3	Rv0491	Mb0501
19	mce2r	Rv0586	Mb0601
20	mmaA4	Rv0642c	Mb0661c
21	mmaA3	Rv0643c	Mb0662c
22	mmaA2	Rv0644c	Mb0663c
23	mmaA1	Rv0645c	Mb0664c
24	sigL	Rv0735	Mb0756
25	phoP	Rv0757	Mb0780
26	phoR	Rv0758	Mb0781
27	phoT	Rv0820	Mb0843
28	phoY2	Rv0821c	Mb0844c
29	ompA	Rv0899	Mb0923
30	pstA1	Rv0930	Mb0953
31	pknDb	Rv0931c	Mb0954c
32	pstS-1	Rv0934	Mb0959
33	ctpV	Rv0969	Mb0994
34	mprA	Rv0981	Mb1007
35	mprB	Rv0982	Mb1008
36	pepD	Rv0983	Mb1009

hsp22.5	Rv0990c	Mb1017c
sigE	Rv1221	Mb1253
lpqY	Rv1235	Mb1267
fadD33	Rv1345	Mb1380
irtA	Rv1348	Mb1383
irtB	Rv1349	Mb1384
	Rv1410c	Mb1445c
lprG	Rv1411c	Mb1446c
pks5	Rv1527c	Mb1554c
lspA	Rv1539	Mb1566
pks10	Rv1660	Mb1688
pks7	Rv1661	Mb1689
pknE	Rv1743	Mb1772
plcD	Rv1755c	Mb1784c
eccD5	Rv1795	Mb1823
mgtC	Rv1811	Mb1841
secA2	Rv1821	Mb1852
modA	Rv1857	Mb1888
katG	Rv1908c	Mb1943c
-	Rv1931c	Mb1966c
tpX	Rv1932	Mb1967
mel2	Rv1936	Mb1971
yrbE3A	Rv1964	Mb1999
-	Rv1979c	Mb2001c
mpt64	Rv1980c	Mb2002c
nrdF1	Rv1981c	Mb2003c
vapC36	Rv1982	Mb2004c
dosT	Rv2027c	Mb2052c
acr1 (hspX)	Rv2031c	Mb2057c
acg	Rv2032	Mb2058
pks12	Rv2048c	Mb2074c
sigC	Rv2069	Mb2095
pafA	Rv2097c	Mb2124c
mpa	Rv2115c	Mb2139c
-	Rv2136c	Mb2160c
ctaC	Rv2200c	Mb2223c
caeA	Rv2224c	Mb2248c
ptpA	Rv2234	Mb2258
kasB	Rv2246	Mb2270
mbtB	Rv2383c	Mb2404c

aprA	Rv2395a	Mb2417a
aprB	Rv2395b	Mb2417b
ahpC	Rv2428	Mb2454
ndk	Rv2445c	Mb2472c
sigA	Rv2703	Mb2722
ideR	Rv2711	Mb2730
clgR	Rv2745c	Mb2766c
-	Rv2869c	Mb2894c
rip	Rv2869c	Mb2894c
fadD26	Rv2930	Mb2955
drrC	Rv2938	Mb2963
fadD28	Rv2941	Mb2966
mmpL7	Rv2942	Mb2967
lppX	Rv2945c	Mb2970c
pks1	Rv2947c	Mb2971c
virS	Rv3082c	Mb3109c
devR	Rv3133c	Mb3157c
nuoG	Rv3151	Mb3175
sigH	Rv3223c	Mb3250c
kefB	Rv3236c	Mb3264c
ctpC	Rv3270	Mb3298
sigF	Rv3286c	Mb3314c
choD	Rv3409c	Mb3443c
sigD	Rv3414c	Mb3448c
whiB3	Rv3416	Mb3450
yrb4A	Rv3501c	Mb3531c
ltp2	Rv3540c	Mb3570c
-	Rv3541c	Mb3571c
-	Rv3542c	Mb3572c
fadE29	Rv3543c	Mb3573c
fadE28	Rv3544c	Mb3574c
cyp125	Rv3545c	Mb3575c
hsaC	Rv3568c	Mb3599c
espD	Rv3614c	Mb3644c
espC	Rv3615c	Mb3645c
-	Rv3654c	Mb3678c
-	Rv3655c	Mb3679c
dppA	Rv3666c	Mb3690c
-	Rv3671c	Mb3695c
ponA2	Rv3682	Mb3707

117	lpqH	Rv3763	Mb3789
118	fbpA	Rv3804c	Mb3834c
119	pirG	Rv3810	Mb3840
120	mmpL8	Rv3823c	Mb3853c
121	espR	Rv3849	Mb3879
122	espF	Rv3865	Mb3895
123	espH	Rv3867	Mb3897
124	eccA1	Rv3868	Mb3898
125	eccB1	Rv3869	Mb3899
126	eccCA1	Rv3870	Mb3900
127	eccCB1	Rv3871	Mb3901
128	PE35	Rv3872	Mb3902
129	PPE68	Rv3873	Mb3903
130	esxB	Rv3874	Mb3904
131	esxB	Rv3874	Mb3904
132	esxA	Rv3875	Mb3905
133	esxA	Rv3875	Mb3905
134	eccD1	Rv3877	Mb3907
135	eccD1	Rv3877	Mb3907
136	mycP1	Rv3883c	Mb3913c
137	mycP1	Rv3883c	Mb3913c

Supplemental Table 2. List of 67 *M. bovis* genomes retrieved from the NCBI public database used in the comparative genomic analysis of *M. bovis* isolates from the Nile Delta.

Used acronym	Accession number	Species	Strain	Contigs	%GC	Isolation date	Country	Host
M.Africanum-Gm041182	NC_015758.1	Mycobacterium tuberculosis var. africanum	GM04118 2	1	65.60	1968	West Africa	*Homo sapiens*
M.Canettii-Cipt-140010059	NC_015848.1	Mycobacterium canittii	CIPT-140010059	1	65.40	1997	Somalia	*Homo sapiens*
M.Caprae_ Allgaeu	NZ_CP01640 1.1	Mycobacterium tuberculosis var. caprae	Allgaeu	1	65.60	1999	Spain	*Sheep, Ovis sp.*
M.Tb_Haarlem_V2	NC_022350.1	Mycobacterium tuberculosis var. tuberculosis	Haarlem	1	65.60	2005	USA	*Homo sapiens*
M.Tb-CDC1551	NC_002755.2	Mycobacterium tuberculosis var. tuberculosis	CDC1551	1	65.60	2002	USA	*Homo sapiens*
M.Tb-H37ra	NC_009525.1	Mycobacterium tuberculosis var. tuberculosis	H37Ra	1	65.60	1934	USA	*Homo sapiens*
M.Tb-H37rv	NC_000962.3	Mycobacterium tuberculosis var. tuberculosis	H37Rv	1	65.60	1934	USA	*Homo sapiens*
Mb-04-303-Argentina.La.Pampa-2004-Wild.Boar	NZ_AVSW00 000000.1	Mycobacterium tuberculosis var. bovis	04-303	169	65.52	2004	Argentina	*Pig, Sus scrofa*
Mb-05-566-Argentina.Buenos.Aires-2000-Cattle	NZ_JPFR000 00000.1	Mycobacterium tuberculosis var. bovis	05-566	89	65.50	2000	Argentina	*Bovine, Bovinae*
Mb-05-567-Argentina.Buenos.Aires-2000-Cattle	NZ_JPFQ000 00000.1	Mycobacterium tuberculosis var. bovis	05-567	69	65.57	2000	Argentina	*Bovine, Bovinae*
Mb-08-08bf2-Brazil.Sao.Paulo.Boituva-2008-Cattle	NZ_JQET000 00000.1	Mycobacterium tuberculosis var. bovis	08-08BF2	108	65.52	2008	Brazil	*Bovine, Bovinae*
Mb-0822-11-Brazil.Sao.Paulo.Porangaba-2012-Cattle	NZ_JQEW00 000000.1	Mycobacterium tuberculosis var. bovis	0822-11	85	65.54	2012	Brazil	*Bovine, Bovinae*
Mb-09-1191-Argentina.Buenos.Aires.Balcarce-2009-Cattle	NZ_JPFP0000 0000.1	Mycobacterium tuberculosis var. bovis	09-1191	86	65.57	2009	Argentina	*Bovine, Bovinae*
Mb-09-1193-Argentina.Buenos.Aires.Trenque.Lauquen-2009-Cattle	NZ_JQEN000 00000.1	Mycobacterium tuberculosis var. bovis	09-1193	89	65.50	2009	Argentina	Bovine, Bovinae
Mb-1595-S.Korea-2015-Cattle	NZ_CP01209 5.1	Mycobacterium tuberculosis var. bovis	1595	1	65.64	2012	Korea	Cattle, *Bos* sp.
Mb-18-08c-Brazil.Paran.Palmeiras-2008-Cattle	NZ_JQEQ000 00000.1	Mycobacterium tuberculosis var. bovis	18-08C	63	65.58	2008	Brazil	Bovine, Bovinae

Mb-32-08-Brazil.Minas.Gerais.Uberlandia-2008-Cattle	NZ_JQER00000000.1	Mycobacterium tuberculosis var. bovis	32-08	83	65.56	2008	Brazil	Bovine, Bovinae
Mb-35-Brazil.Mato.Grosso.Do.Sul.Dois.Irmaos.Do.Buriti-2003-Cattle	NZ_JQEV00000000.1	Mycobacterium tuberculosis var. bovis	35	94	65.60	2003	Brazil	Bovine, Bovinae
Mb-45-08b-Brazil.Minas.Gerais.Arax-2008-Cattle	NZ_JQEP00000000.1	Mycobacterium tuberculosis var. bovis	45-08B	72	65.55	2008	Brazil	Bovine, Bovinae
Mb-49-09-Brazil.Sao.Paulo.Presidente.Venceslau-2009-Cattle	NZ_JQES00000000.1	Mycobacterium tuberculosis var. bovis	49-09	61	65.59	2009	Brazil	Bovine, Bovinae
Mb-50-Brazil.Acre.Tarauac-Sep2014-Cattle	NZ_JQEU00000000.1	Mycobacterium tuberculosis var. bovis	50	84	65.55	2009	Brazil	Bovine, Bovinae
Mb-534-Argentina.Santa.Fe.Las.Colonias-1994-Cattle	NZ_JQEM00000000.1	Mycobacterium tuberculosis var. bovis	534	72	65.56	1994	Argentina	Bovine, Bovinae
Mb-61-09-Brazil.Sao.Paulo.Novo.Castilho-2009-Sheep	NZ_JQEX00000000.1	Mycobacterium tuberculosis var. bovis	61-09	68	65.59	2009	Brazil	Sheep, *Ovis* sp.
Mb-Af2122.97	NC_002945.3	Mycobacterium tuberculosis var. bovis	AF2122/97	1	65.60	1997	UK	Bovine, Bovinae
Mb-An5-Brazil-2014-Cattle	NZ_AWPL00000000.1	Mycobacterium tuberculosis var. bovis	AN5	70	65.60	1948	UK	Cattle, *Bos* sp.
Mb-Atcc-Baa-935-Usa-Sep2014	NZ_CP009449.1	Mycobacterium tuberculosis var. bovis	BAA-935	1	65.61	1997	UK	Cattle, *Bos primigenius*
Mb-B-3222-S.Korea.Hongseong-2013-Cattle	NZ_LNOF00000000.1	Mycobacterium tuberculosis var. bovis	B-3222	56	65.60	2018	S.Korea	*Cattle, Bos taurus*
Mb-B2.7505-Uganda-Oct2006-Human	NZ_JKAL00000000.1	Mycobacterium tuberculosis var. tuberculosis	B2.7505	243	65.50	2006	Uganda	Human, *Homo sapiens*
Mb-Bcg-Atcc-35743-Beijing-2011	NZ_CP003494.1	Mycobacterium tuberculosis var. bovis	BCG-63839	1	65.70	2011	Beijing	*Elk, Cervus canadensis*
Mb-Bcg-Mexico1931-2010	NC_016804.1	Mycobacterium tuberculosis var. bovis - BCG	BCG-Mexico1931	1	65.70	2010	Mexico	*Cattle, Bos sp.*
Mb-Bcg-Moreau-Rdj-2016	NZ_AM412059.1	Mycobacterium tuberculosis var. bovis - BCG	BCG-Moreau-RDJ	1	65.70	2016	Brazil	*Cattle, Bos taurus*
Mb-Bcg-Pasteur1173p2-2007	NC_008769.1	Mycobacterium tuberculosis var. bovis - BCG	BCG-Pasteur1173P2	1	65.70	2007	France	*Deer, Odocoileus virginianus*
Mb-BCG-Russia-2015	NZ_CP013741.1	Mycobacterium tuberculosis var. bovis - BCG	BCG-Russia	1	65.70	2015	Russia	*Deer, Odocoileus virginianus*
Mb-Bcg-Tokyo172-2008	NZ_CP014566.1	Mycobacterium tuberculosis var. bovis - BCG	BCG-Tokyo172	1	65.70	2008	Tokyo	*Deer, Odocoileus virginianus*

Mb-Bcg3281-China-2015	NZ_CP008744.1	Mycobacterium tuberculosis var. bovis - BCG	BCG3281	1	65.70	2015	China	*Cattle, Bos taurus*
Mb-Bcg368-Russia-2014	NZ_CP009243.1	Mycobacterium tuberculosis var. bovis - BCG	BCG368	1	65.70	2014	Russia	*Cattle, Bos taurus*
Mb-Bz.31150-Uganda-Jun2007-Human	NZ_JKAM00000000.1	Mycobacterium tuberculosis var. bovis	Bz.31150-Uganda	141	65.50	2007	Uganda	Human, *Homo sapiens*
Mb-MB1-Spain-2015-Cattle	NZ_CDHF00000000.1	Mycobacterium tuberculosis var. bovis	MB1	47	65.50	2015	Spain	*Cattle, Bos taurus*
Mb-Mb3-Spain-2015-Wild.Boar	NZ_CDHH00000000.1	Mycobacterium tuberculosis var. bovis	MB3	88	65.50	2015	Spain	*Pig, Sus scrofa*
Mb-Mb4-Spain-2015-Wild.Boar	NZ_CDHE00000000.1	Mycobacterium tuberculosis var. bovis	MB4	109	65.50	2015	Spain	*Pig, Sus scrofa*
Mb-Mburu-001-Uruguay.San.Jose-2014-Cattle	NZ_LFGY00000000.1	Mycobacterium tuberculosis var. bovis	MbURU-001	100	65.50	2014	Uruguay	*Cattle, Bos taurus*
Mb-Sp38-Brazil.Sao.Paulo-2015	NZ_CP015773.1	Mycobacterium tuberculosis var. bovis	SP38	1	65.50	2015	Brazil	
Mb-W-1171-S.Korea-Jun2014-Wild.Boar	JXTK00000000	Mycobacterium tuberculosis var. bovis	W-1171	50	65.50	2014	S.Korea	*Pig, Sus scrofa*

7

Supplemental Table 3. List of 27 SNPs predicted to be uniquely associated with MBE4 used for genotype comparison between MBE4, MBE5 and MBE7 relative to the reference virulence strain of *M. bovis*.

SNPs uniquely present in MBE4

#	Reference position	Reference allele	MBE4	MBE5	MBE7	Annotation	SNP type	Amino acid change
1	339620	C	G	C	C	BQ2027_MB0287c / PE_PGRS4	synonymous	
2	1165589	T	C	T	T	BQ2027_MB1071c	non-synonymous	Glu129Gly
3	1619153	A	G	A	A	BQ2027_MB1476c / PE_PGRS26	synonymous	
4	1619361	T	G	T	T		non-synonymous	Asn354Thr
5	2283587	G	T	G	G	BQ2027_MB2074c / pks12	non-synonymous	Asp3908Glu
6	2283590	G	A	G	G		synonymous	
7	2283596	G	C	G	G		non-synonymous	Phe3905Leu
8	2283598	A	G	A	A		non-synonymous	Phe3905Leu
9	2283607	A	G	A	A		non-synonymous	Phe3902Leu
10	2283616	T	G	T	T		non-synonymous	Thr3899Pro
11	3082946	T	A	T	T	intergenic		
12	3836507	T	C	T	T	BQ2027_MB3495	synonymous	
13	3878929	G	C	G	G	BQ2027_MB3537 / PE_PGRS53	non-synonymous	Lys775Asn
14	3878937	C	A	C	C		non-synonymous	Ala778Asp
15	3878946	T	A	T	T		non-synonymous	Ile781Asn
16	3878985	G	C	G	G		non-synonymous	Gly794Ala
17	3884294	A	G	A	A	BQ2027_MB3538 / PE_PGRS54	non-synonymous	Asp1106Gly
18	3884519	C	T	C	C		non-synonymous	Ala118Val
19	3890762	T	C	T	T	BQ2027_MB3541 / PE_PGRS55	synonymous	
20	3890767	G	C	G	G		non-synonymous	Gly834Ala
21	3893080	A	G	A	A		non-synonymous	Asp1605Gly
22	4259281	T	C	T	T	intergenic		

SNPs uniquely absent in MBE4	1	928975	G	G	A	A	BQ2027_ MB0857C / PE_PGRS 14		
	2	928981	G	G	A	A			
	3	928985	A	A	T	T			
	4	928987	G	G	A	A			
	5	928991	A	A	G	G			

12

Supplemental Table 4. List of predicted insertions/deletions (InDels) used for genotype comparison between MBE4, MBE5 and MBE7 relative to the reference virulence strain of *M. bovis*.

Region	*Type*	*Length*	*Variant ratio*	*Non-synonymous*	*Coding region change*[*]	*Amino acid change*	*Isolate*
336965..336973	Deletion	9	0.96	Yes	PE_PGRS4-BQ2027_MB0287C:c.2359_2367delCCGGCGGCA	PE_PGRS4-BQ2027_MB0287C:p.Pro793_Ala795del	MBE4
922111..922216	Deletion	106	0.98	Yes	PE_PGRS14-BQ2027_MB0857C:c.2419_2524delCGGCACCGGCTTCGGGGCTAACACCGGCGGCAACGGCGGCGACACGACGATAGTTGTCAACGGCATGCTCGCCCCGTCTACCCTCGGCTACGGTGGCAACGGCGCC	PE_PGRS14-BQ2027_MB0857C:p.His808fs	MBE4
3490234^3490235	Insertion	3	0.93	Yes	moeB1-BQ2027_MB3231C:c.588_589insCCG	moeB1-BQ2027_MB3231C:p.Pro196_Gly197insPro	MBE4
1696886..1696893	Deletion	8	0.98	Yes	pks5-BQ2027_MB1554C:c.4344_4351delGATCGAGG	pks5-BQ2027_MB1554C:p.Glu1448fs	MBE4, MBE5, MBE7
1792710..1792714	Deletion	5	1.00	Yes	cya-BQ2027_MB1651C:c.454_458delCTAGC	cya-BQ2027_MB1651C:p.Leu152fs	MBE4, MBE5, MBE7
2003322..2003448	Deletion	127	1.00	Yes	PPE29-BQ2027_MB1829:c.1050_1176delGCAGGCGACGCGCCTGGCCGCCGCGCCGATTGCGGCCACCGACGGCGCGGGTGCGTTGCTCGGTGGCATGGCGCTGTCGGGCTTGGCTGGCCGCGCTGCCGCCGGTTCTACCGGCCACCCCATCGGC	PPE29-BQ2027_MB1829:p.Gln351fs	MBE4, MBE5, MBE7
2034192..2034247	Deletion	56	0.87	Yes	BQ2027_MB1860:c.494_495delAG	BQ2027_MB1860:p.*165fs	MBE4, MBE5, MBE7
2034270..2034325	Deletion	56	0.79	-			MBE4, MBE5, MBE7
2278028..2278038	Deletion	11	1.00	Yes	BQ2027_MB2107C:c.305_315delTGGGGGGGGGG	BQ2027_MB2107C:p.Val102fs	MBE4, MBE5, MBE7
3030578..3030650	Deletion	73	0.97	-			MBE4, MBE5, MBE7
3080417..3080470	Deletion	54	0.74	Yes	BQ2027_MB2884C:c.126_179delTGTGTCCGATGGTGGCGACCCGCTGCGCCCGGCTTCGCCGCGCTTGCGATCGCC	BQ2027_MB2884C:p.Val43_Pro60del	MBE4, MBE5, MBE7
3080522..3080575	Deletion	54	0.74	Yes	BQ2027_MB2884C:c.21_74delGTCCGATGGTGGCGACCCGCTGCGCCCGGCTTCGCCGCGCTTGCGATCGCCTGT	BQ2027_MB2884C:p.Val43_Pro60del	MBE4, MBE5, MBE7
3236614..3236625	Deletion	12	0.99	Yes	BQ2027_MB2997C:c.269_280delCCCGCGACGACA	BQ2027_MB2997C:p.Thr90_Asp93del	MBE4, MBE5, MBE7
803772..803786	Deletion	15	0.96	Yes	rplV-BQ2027_MB0726:c.529_543delGCGCCCGCCAAGAAG	rplV-BQ2027_MB0726:p.Lys181_Lys185del	MBE4, MBE5, MBE7
958750..958773	Deletion	24	0.88	Yes	rpfA-BQ2027_MB0891C:c.809_832delCCGCCGACCTGGCGCCACCCGCGC	rpfA-BQ2027_MB0891C:p.Asp280_Ala287del	MBE4, MBE5, MBE7
1377637^1377638	Insertion	68	1.00	Yes	PE_PGRS23-BQ2027_Mb1275C:c.723_724insTGGGGCAACGGGGGGGCGGGTGGAGCCGGAGCGGCAGGAGCGGCCGGCGCCAACGGGCAAAACCCGGT	PE_PGRS23-BQ2027_Mb1275C:p.Val242fs	MBE4, MBE5, MBE7
1829237^1829238	Insertion	90	0.99	Yes	PE_PGRS30-BQ2027_MB1679C:c.1297_1298insGAGCCGGCGGGGCCGGCGGGGCCGGCGGCGTCGGCGGGCTGCTCTACGGCAACGGCGGGAACGGGGGGGCCGGTGGCATCGGCGGGGCTG	PE_PGRS30-BQ2027_MB1679C:p.Ser432_Ala433insGlyAlaGlyGlyAlaGlyGlyAlaGlyGlyValGlyGlyLeuLeuTyrGlyAsnGlyGlyAsnGlyGlyAlaGlyGlyIleGlyGlyAla	MBE4, MBE5, MBE7
1920599^1920600	Insertion	57	0.81	Yes	BQ2027_MB1757C:c.32_33insGACGCCCGGTGACGATGCGCGCCGTTTGCGGCGCGAGGAGGAACCGGGCAATCAGAC	BQ2027_MB1757C:p.Thr11_Arg12insThrProGlyAspAspAlaArgArgLeuArgArgGluGluGluProGlyAsnGlnThr	MBE4, MBE5, MBE7
1920633^1920634	Insertion	57	0.87	-			MBE4, MBE5, MBE7
2006348^2006349	Insertion	106	1.00	Yes	PE_PGRS32b-BQ2027_MB1831C:c.370_371insCGCCCGGGGGTGCGTCGGCAGGCTTCGATGGGGGCGCCGGCGGAAACGGGGGTTCGGGTGGTCGCGGTGGACTGTTGTTCGGCAACGGCGGCAACGGCTCGGTCGG	PE_PGRS32b-BQ2027_MB1831C:p.Val124fs	MBE4, MBE5, MBE7
2279581^2279582	Insertion	174	1.00	Yes	BQ2027_MB2108:c.1036_1037insCCCGACAGCGCGCCGACCCCCAGCCCGGCACCGATCGCGCCACCAACCACCGACAACGCCAGCGCCATGACACCCATCGCGCCCATGGTCGCTAATGGCCCGCCAGCATCCCCCGCCCCCCCGGCCCCCACCCCCGCGGGGCCACTGCCCGCCTACGGCGCCGACCTGCGCCCA	BQ2027_MB2108:p.Pro346_Gly347insArgGlnArgAlaAspProGlnProGlyThrAspArgAlaThrAsnHisArgGlnArgGlnArgHisAspThrHisArgAlaHisGlyArg*TrpProAlaSerIleProArgProProGlyProHisProArgGlyAlaThrAlaArgLeuArgArgArgProAlaProThr	MBE4, MBE5, MBE7
2286979^2286980	Insertion	57	0.70	Yes	BQ2027_MB2117:c.22_23insGTGGCGACCCGCCGCACCCGGCTCCGCCGCGCTTGCGATCGCCACTGGACCCAACAA	BQ2027_MB2117:p.Pro30_Leu31insProHisProAlaProProArgLeuArgSerProLeuAspProThrSerGlyAspPro	MBE4, MBE5, MBE7
2287084^2287085	Insertion	57	0.66	Yes	BQ2027_MB2117:c.127_128insACCCAACAAGTGGCGACCCGCTGCACCCGGCTCCGCCGCGCTTGCGATCGCCACTGG	BQ2027_MB2117:p.Leu42_Val43insAspProThrSerGlyAspProLeuHisProAlaProProArgLeuArgSerProLeu	MBE4, MBE5, MBE7
2339696^2339697	Insertion	116	0.72	-			MBE4, MBE5, MBE7
2339798^2339799	Insertion	116	0.76	-			MBE4, MBE5, MBE7
2497996^2497997	Insertion	2	0.98	Yes	sseB-BQ2027_MB2314:c.83_84insTG	sseB-BQ2027_MB2314:p.Ala29fs	MBE4, MBE5, MBE7
2801004^2801005	Insertion	80	1.00	-			MBE4, MBE5, MBE7
3016328^3016329	Insertion	3	0.93	Yes	BQ2027_MB2822:c.109_110insTGG	BQ2027_MB2822:p.Val36_Gly37insVal	MBE4, MBE5, MBE7
4116248^4116249	Insertion	59	0.86	-			MBE4, MBE5, MBE7
4116299^4116300	Insertion	59	0.82	-			MBE4, MBE5, MBE7
424231^424232	Insertion	102	0.94	No	hspR-BQ2027_MB0361:c.373_374insGCCGGTGAGCGAGCGCGCGTAGCGGGGGAGCGAACGGCGCAGTTGGCACCAGCCGGTGAGCGAGCGCGCGTAGCGGGGGAGCGAACGGCGCAGTTGGCACCA		MBE4, MBE5, MBE7
4268223^4268224	Insertion	60	1.00	Yes	BQ2027_MB3865:c.1038_1039insAACGGCCAGCCGCCGGTACCCCCACCCGACATTCCCGAGGAGCGGCTCAACCTGCCGCCG	BQ2027_MB3865:p.Ile346_Ile347insAsnGlyGlnProProValProProProAspIleProGluGluArgLeuAsnLeuProPro	MBE4, MBE5, MBE7

959103^959104	Insertion	23	0.91	Yes	rpfA-BQ2027_MB0891C:c.478_479insGCCCGCCGACCTGGCGCCACCCG	rpfA-BQ2027_MB0891C:p.Ala160fs	MBE4, MBE5, MBE7
963615^963616	Insertion	169	0.99	Yes	PE_PGRS15-BQ2027_MB0896C:c.512_513insCGGAGGGGCCGGCGCGGTCGGCGGCGTCGGCGGCAACGGGGGGTGGTTATACGGCAAGGGCGGGGCCGGCGGGCTCGGGGGGACCGGGGTGGCCGGCGTCAATGGCGGTATGGGCGCAGCAGGAGGTGCCGGCGGCAACGCCTACCTGTTCGGTTCTGGCGGCGCGGGC	PE_PGRS15-BQ2027_MB0896C:p.Arg173fs	MBE4, MBE5, MBE7
1025280..1025282	Deletion	3	0.97	Yes	mntH-BQ2027_MB0948C:c.1237_1239delCTG	mntH-BQ2027_MB0948C:p.Leu413del	MBE5
1629869..1629921	Deletion	53	0.71	-			MBE5
1743072..1743275	Deletion	204	0.96	-			MBE5
1743073..1743275	Deletion	203	0.96	-			MBE5
1743112..1743275	Deletion	164	1.00	-			MBE5
1947030..1947039	Deletion	10	1.00	Yes	PPE24-BQ2027_MB1782C:c.2478_2487delGCCCCAAATC	PPE24-BQ2027_MB1782C:p.Pro830fs	MBE5
2963270..2963404	Deletion	135	0.71	Yes	PE_PGRS47-BQ2027_MB2761:c.600_734delGGCCGCCGGCCCCGGCCGGGCCGGGGGGGGGGTGGGGGGGGGCCGAGGCGGCGGCGTAGGCGGCGCCGCCCTCGGCGGCGGCGCCCAGGCCGCCGGTGGCAACGGCGGGGCGGGCGGGGGCGGGGGGCGGTTCGG	PE_PGRS47-BQ2027_MB2761:p.Ala202_Ala246del	MBE5
3080423..3080476	Deletion	54	0.81	Yes	BQ2027_MB2884C:c.120_173delATCGCCTGTGTCCGATGGTGGCGACCCGCTGCGCCCGGCTTCGCCGCGCTTGCG	BQ2027_MB2884C:p.Val43_Pro60del	MBE5
3898646..3898654	Deletion	9	0.75	Yes	PE_PGRS53-BQ2027_MB3537:c.2498_2506delACGGCGGCG	PE_PGRS53-BQ2027_MB3537:p.Asp836_Gly838del	MBE5
958857..959042	Deletion	186	1.00	Yes	rpfA-BQ2027_MB0891C:c.540_725delCGTACGCCCTGCCTGCGCCGCTGGGGCTGGAGTGCTCCCGGTAGCCCCCGCCGACCTGGCGCCACCCGCGTCAACGGCGAACCAGCACCGCTGGCCCCGCCGCCCGCCGACCGTGGCGCCCCCCGCGCCCGCCGGCCTGGCGCCCCCCGCGCCCGCCGCCCGGGCCCCGCCCGTGGACCTTGCCGT	rpfA-BQ2027_MB0891C:p.Val181_Val242del	MBE5
1197528^1197529	Insertion	17	0.99	Yes	pra-BQ2027_MB1107:c.33_34insCCACCGCCCCCGCCACC	pra-BQ2027_MB1107:p.Ala12fs	MBE5
1332211^1332212	Insertion	15	0.95	Yes	PPE18-BQ2027_MB1228:c.1057_1058insTGCTGGGCGGGCTGC	PPE18-BQ2027_MB1228:p.Leu357_Pro358insLeuLeuGlyGlyLeu	MBE5
2994841^2994842	Insertion	24	0.98	Yes	BQ2027_MB2801C:c.366_367insGTGATGGATGTCTACTTTCTGGCC	BQ2027_MB2801C:p.Gln122_Gly123insValMetAspValTyrPheLeuAla	MBE5
3025862^3025863	Insertion	2	0.99	Yes	BQ2027_MB2835:c.590_591insGT	BQ2027_MB2835:p.Gly198fs	MBE5
333407^333408	Insertion	22	1.00	-			MBE5
3436664^3436665	Insertion	3	0.93	Yes	PPE53-BQ2027_MB3183C:c.177_178insGCG	PPE53-BQ2027_MB3183C:p.Ala63_Met64insAla	MBE5
4116257^4116258	Insertion	59	0.87	-			MBE5
55529^55530	Insertion	3	0.93	Yes	ponA1-BQ2027_MB0051:c.1883_1884insGCC	ponA1-BQ2027_MB0051:p.Pro632_Ser633insPro	MBE5
993298..993300	Deletion	3	1.00	-			MBE5, MBE7
1276944^1276945	Insertion	72	0.72	Yes	BQ2027_MB1188C:c.642_643insGCGCCGGCCTCGGCGGCACCGGCGCCAGCGGCCGCTCCGGCGCCTGCCTCGGGCTTCGGCGCCGACGCTCCG	BQ2027_MB1188C:p.Pro214_Pro215insAlaProAlaSerAlaAlaProAlaProAlaAlaAlaProAlaProAlaSerGlyPheGlyAlaAspAlaPro	MBE5, MBE7
1349220^1349221	Insertion	5	0.99	Yes	PE14-BQ2027_MB1246C:c.203_204insACAAG	PE14-BQ2027_MB1246C:p.Pro69fs	MBE5, MBE7
2725747^2725748	Insertion	24	1.00	Yes	PE_PGRS43a-BQ2027_MB2518C:c.673_674insAGGATGGGGCGGCTGGTGTTGCCG	PE_PGRS43a-BQ2027_MB2518C:p.Arg225delinsGlnAspGlyAlaAlaGlyValAlaGly	MBE5, MBE7
3157303^3157304	Insertion	13	0.97	Yes	ppsA-BQ2027_MB2956:c.2853_2854insCACACCCCCCACC	ppsA-BQ2027_MB2956:p.Pro952fs	MBE5, MBE7
3247049^3247050	Insertion	9	0.96	Yes	ddlA-BQ2027_MB3005C:c.14_15insCCGGCGTGA	ddlA-BQ2027_MB3005C:p.Arg7_Val8insAspArgArg	MBE5, MBE7
3724030^3724031	Insertion	33	1.00	Yes	PPE55b-BQ2027_MB3379C:c.7424_7425insCCGTCGTCGGGCTTCTTCAACACCGGGGCGGGC	PPE55b-BQ2027_MB3379C:p.Val2475_Gly2476insArgArgArgAlaSerSerThrProGlyArgAla	MBE5, MBE7
3796794^3796795	Insertion	116	0.78	Yes	BQ2027_MB3434:c.2349_2350insCTTTAGCCCGGTGACGATACGGGCCGCGTAGCGGCCCGAGGAGGAGCCGGGCAATCGGCTTAGCCCGGTGACGATACGGGCCGCGTAGCGGCCCGAGGAGGAGCCGGGCAATCGGC	BQ2027_MB3434:p.Ala785fs	MBE5, MBE7
1629756..1629808	Deletion	53	0.66	Yes	BQ2027_MB1493C:c.1_12delGTGAACCGCGCC	BQ2027_MB1493C:p.Val1_Ala4del	MBE7
2034189..2034244	Deletion	56	0.86	Yes	BQ2027_MB1860:c.491_495delCCTAG	BQ2027_MB1860:p.Thr164fs	MBE7
3027892..3028001	Deletion	110	0.94	-			MBE7
3080425..3080478	Deletion	54	0.84	Yes	BQ2027_MB2884C:c.118_171delCGATCGCCTGTGTCCGATGGTGGCGACCCGCTGCGCCCGGCTTCGCCGCGCTTG	BQ2027_MB2884C:p.Val43_Pro60del	MBE7
1197527^1197528	Insertion	17	0.99	Yes	pra-BQ2027_MB1107:c.32_33insCCCACCGCCCCCGCCAC	pra-BQ2027_MB1107:p.Ala12fs	MBE7
1848834^1848835	Insertion	3	0.94	Yes	BQ2027_MB1757C:c.32_33insGACGCCCGGTGACGATGCGCGCCGTTTGCGGCGCGAGGAGGAACCGGGCAATCAGAC	BQ2027_MB1757C:p.Thr11_Arg12insThrProGlyAspAspAlaArgArgLeuArgArgGluGluGluProGlyAsnGlnThr	MBE7

*Codon region changes: fs: frame-shift, del: deletion, ins: insertion

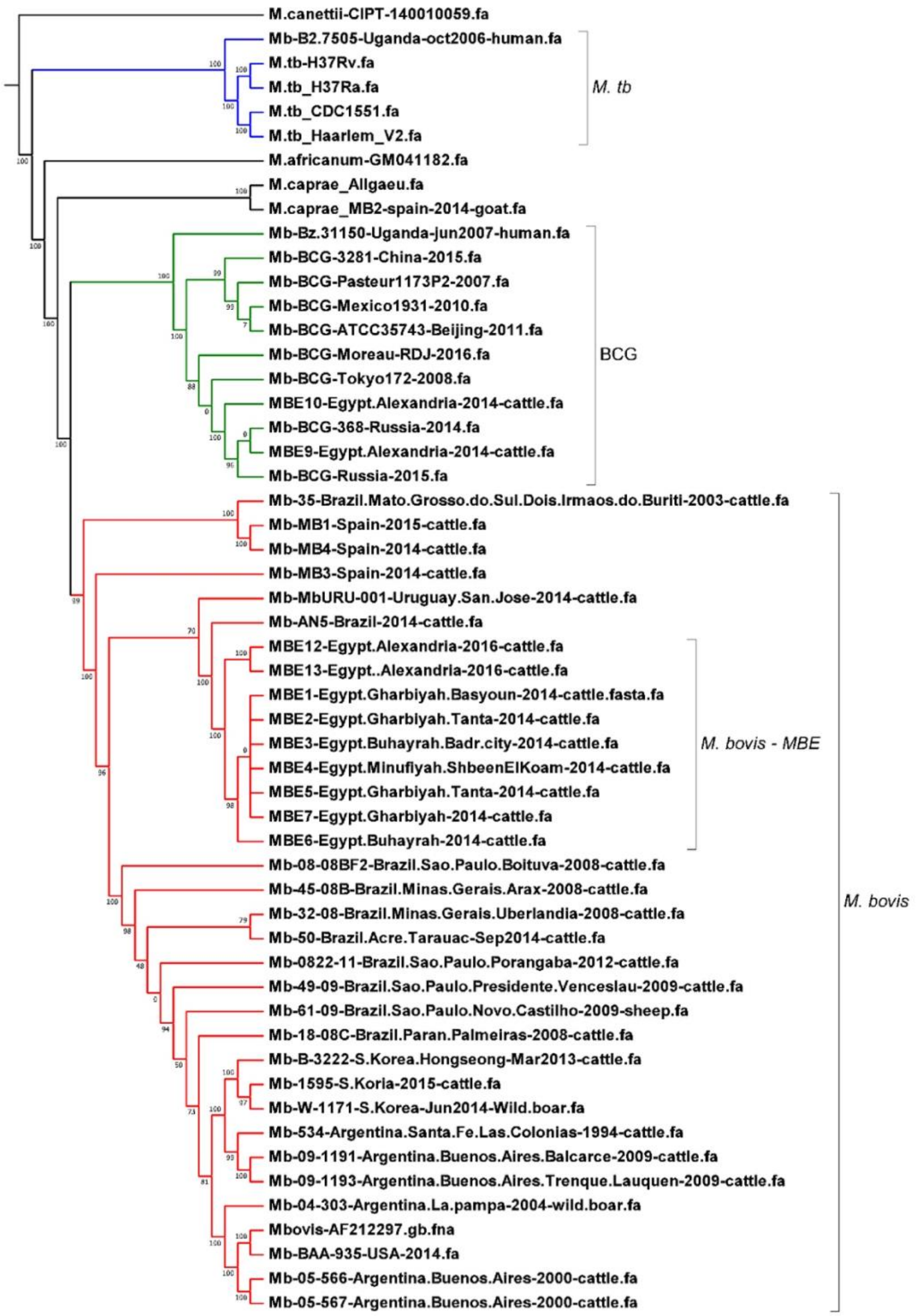

Supplemental Fig. 1. Phylogenetic tree of MBE isolates and an archive of 67 sequenced *M. bovis* genomes. Whole genome sequences were analyzed with the Jukes-Cantor or generalized time-reversible (GTR) models of nucleotide evolution using Harvest software, and the tree was visualized with MEGA7, each strain identified by ID, origin of isolation, year of isolation, and species of isolation.

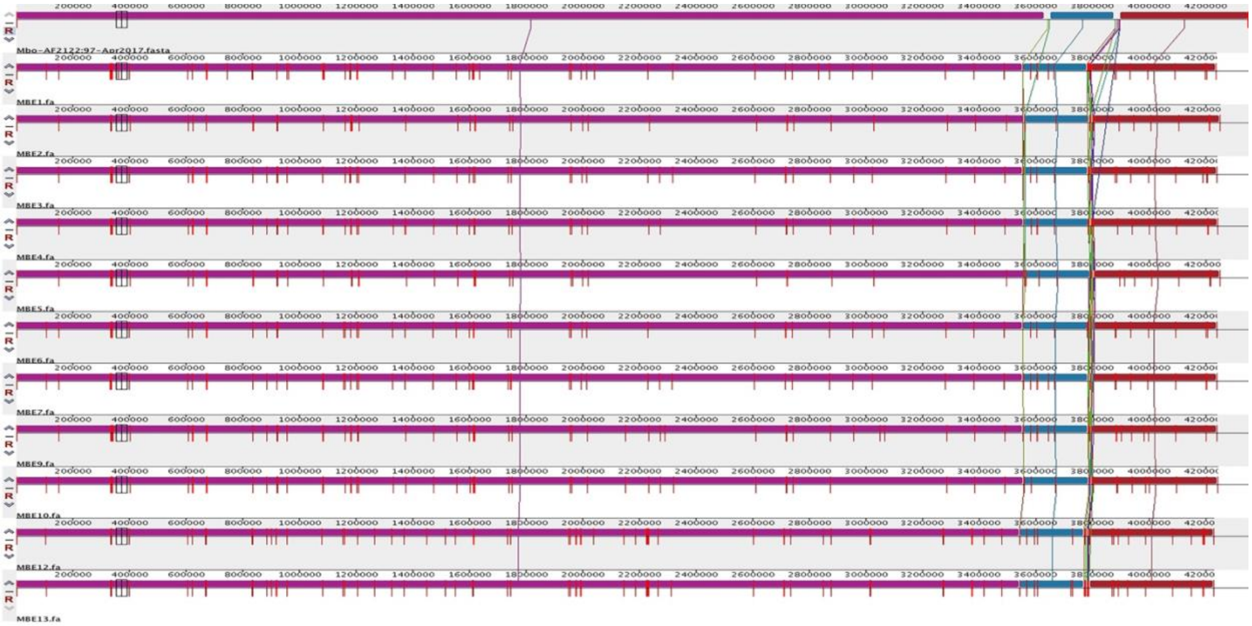

Supplementary Fig. 2. Core-genome multiple alignment for all the *M. bovis* isolates from Nile delta, MAUVE alignment showing no major genomic rearrangements.

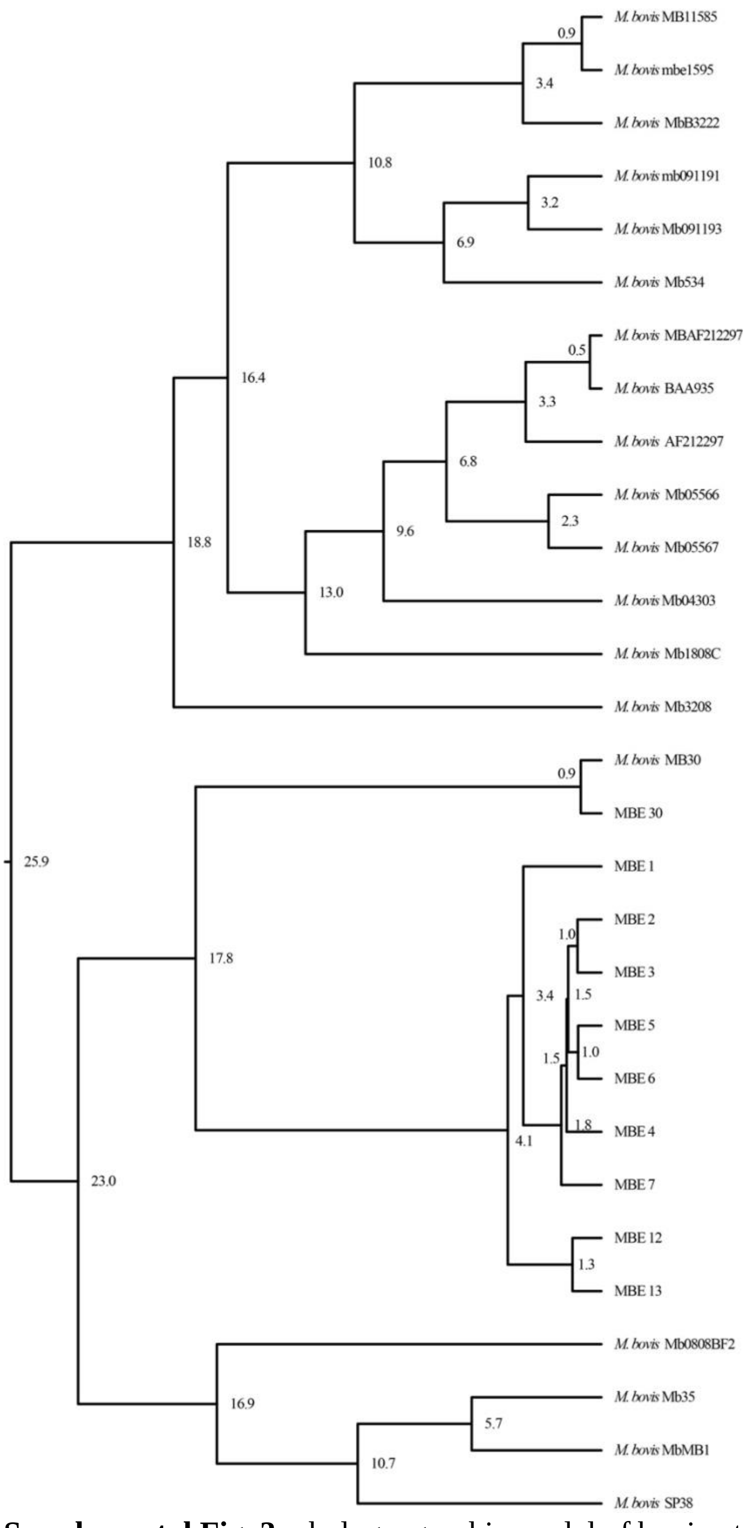

Supplemental Fig. 3. phylogeographic model of bovine tuberculosis in the Nile Delta.

References.

1. Forrellad, M. A. *et al.* Virulence factors of the Mycobacterium tuberculosis complex. doi:10.4161/viru.22329
